# Self‐Assembled *Dictamni Cortex* Nanoparticles Ameliorate Psoriasis by Epigenetic Modulation of HSP90AB1 and Suppression of the Inflammatory Response

**DOI:** 10.1002/advs.202512422

**Published:** 2025-10-20

**Authors:** Zhengyi Zhang, Wenqian Du, Xiaojiang Zhang, Hongbo Cui, Baochen Cheng, Xiao Han, Ke He, Tingyi Yin, Xinyi Liu, Ningyi Xian, Ziyang Wang, Meng Liu, Dan Han, Jiankang Liu, Yan Zheng, Ya Wang

**Affiliations:** ^1^ Department of Dermatology The First Affiliated Hospital of Xi'an Jiaotong University Xi'an Shaanxi 710061 China; ^2^ Center for Gut Microbiome Research, Med‐X Institute The First Affiliated Hospital of Xi'an Jiaotong University Xi'an Shaanxi 710061 China; ^3^ College of Chemistry and Materials Science Northwest University Xi'an Shaanxi 710127 China; ^4^ Department of Pathogen Biology School of Basic Medical Science, Xi'an Medical University Xi'an Shaanxi 710021 China; ^5^ Department of Dermatology Tangdu Hospital, Fourth Military Medical University Xi'an Shaanxi 710032 China; ^6^ School of Life Sciences and Health University of Health and Rehabilitation Sciences Qingdao Shandong 266071 China

**Keywords:** Dictamni cortex, HSP90AB1, inflammation, nanotherapy, psoriasis

## Abstract

Psoriasis, a chronic immune‐mediated dermatological disease with high recurrence rates and limited therapeutic efficacy, requires novel treatment strategies. Dictamni Cortex (BXP), a traditional Chinese medicine (TCM), has demonstrated potential in alleviating psoriasis; however, its clinical application is hampered by poor water solubility and low bioavailability. It is developed infinite coordination polymer nanoparticles (BXP‐Fe (III) ICPs, NB), which enhance the aqueous solubility by 95‐fold and bioavailability of BXP, exerting therapeutic effect through efficient transdermal delivery. NB significantly suppresses keratinocyte hyperproliferation, inflammation, and oxidative stress in both M5 (a cocktail of cytokines)‐treated human epidermal keratinocytes (HEKa) cells and imiquimod (IMQ)‐induced psoriatic mice. Nascent proteomics identified heat shock protein 90 alpha family class B member 1 (HSP90AB1) as a key target downregulated by NB. It is further revealed that NB suppresses HSP90AB1 transcription by inhibiting its activator, CCCTC‐binding factor (CTCF), and disrupts the HSP90AB1‐CDC37 (cell division cycle 37, the co‐chaperone) chaperone complex, thereby inactivating the pivotal client proteins STAT3 and Akt. Notably, NB demonstrated superior therapeutic efficacy over the canonical HSP90 inhibitor AUY922. This study highlights NB as a promising topical nanotherapy for psoriasis, integrating TCM with modern nanotechnology to overcome pharmacological limitations. The underlying molecular mechanisms of NB are elucidated through the CTCF‐HSP90AB1‐STAT3 axis.

## Graphical abstract

This study develops self‐assembled Dictamni Cortex (BXP)‐Fe(III) infinite coordination polymer nanoparticles (NB) to improve BXP's solubility and bioavailability for psoriasis. NB transdermally treats psoriasis by targeting the epigenetic regulator CTCF (CCCTC‐binding factor), suppressing HSP90AB1 (heat shock protein 90 alpha family class B member 1) transcription, and disrupting the HSP90AB1‐CDC37 (cell division cycle 37) chaperone complex to inactivate its client proteins. This ultimately attenuates hyperproliferation and inflammation of keratinocytes.

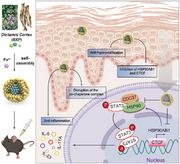

## 1 Introduction

Psoriasis is an immune‐mediated dermatological disorder characterized by complex interactions between innate and adaptive immune systems, with a global prevalence of 2%–3%.^[^ ^1^ ^]^ Histopathological features include the hyperproliferation and abnormal differentiation of keratinocytes, inflammatory infiltration and angiogenesis.^[^ ^2^ ^]^ Notably, psoriasis has a recurrence rate of 98.4%, and its progression is often accompanied by chronicity, disfigurement, disability, and comorbidities, substantially impairing patients’ physical and psychological health and imposing a severe societal and individual burden.^[^ ^3^, ^4^ ^]^ However, current therapeutic strategies are limited by high costs, adverse effects (including immunosuppression, infections, and malignancies), and diminished efficacy with long‐term use.^[^ ^5^, ^6^ ^]^ Topical administration is the preferred approach for the therapy of psoriasis like corticosteroids,^[^ ^7^ ^]^ while due to the skin structure and the molecular weight of topical drugs, the transdermal absorption is low.^[^ ^8^ ^]^

*Dictamni Cortex* (白鲜皮, Baixianpi, BXP), the dried root bark of *Dictamnus dasycarpus*, has been commonly prescribed for skin diseases in TCM history for its dispelling pathogenic wind and relieving itching therapeutic effects. BXP is included in up to 38.36% of patent data of TCM compounds for psoriasis treatment, which was also the most prescribed Chinese herb from the National Health Insurance Research Database in Taiwan.^[^ ^9^, ^10^ ^]^ Although multiple components of BXP have demonstrated anti‐inflammatory, antimicrobial and anti‐allergic properties,^[^ ^11^ ^]^ the in vivo functions of the whole BXP extract and its precise mechanisms against psoriasis remain poorly understood. BXP is a mixture of multiple pharmacological components which could be classified as flavonoid (luteolin, quercetin, wogonin, etc.), terpenoids (fraxinellone, obacunone, etc.), alkaloids (dictamnine, etc.), ceramides, and fatty acid derivatives through HPLC‐MS. The therapeutic potential of BXP in psoriasis is not attributable to individual components alone. Indeed, experimental combinations of its major constituents (luteolin, quercetin, wogonin, fraxinellone, obacunone, dictamnine) at exact ratios and concentrations mimicking those in the native extract failed to recapitulate the efficacy to the whole BXP extracts, aligning with previous study that positive interactions between components of whole plant extracts may explain why crude extracts are often more effective than isolated constituents at an equivalent dose.^[^ ^12^, ^13^ ^]^ However, the poor solubility and low bioavailability of BXP significantly reduced therapeutic effect in vivo. In addition, long‐term oral administration of BXP may induce hepatotoxicity and nephrotoxicity. To solve this, we developed infinite coordination polymer nanoparticles (BXP‐Fe (III) ICPs, NB) by coordinating BXP with Fe^3+^ for psoriasis treatment. Carrier‐free supramolecular nanoparticles formed by coordination‐induced self‐assembly of drug molecules as ligands and metal ions as central atoms have been proven to enhance the solubility and bioavailability of natural products from herbal medicine.^[^ ^14^ ^]^ These nanoparticles, known as ICP, offer high drug loading, adjustable size, pH‐responsive drug release, and good tissue penetration. Structurally, flavonoids with phenolic hydroxyl and ketone groups and terpenoids with ketone groups could coordinate with Fe^3+^ to drive self‐assembly into nanoparticles. Dictamnine, an alkaloid, has an oxygen atom on its furan ring that also coordinates with Fe^3+^. Taken together, their ability to coordinate with Fe^3+^ ensures their involvement in nanoparticle self‐assembly. The coordination not only enhances the aqueous solubility (by 95‐fold) of BXP but also replenishes epidermal iron, which is notably reduced in the epidermis of psoriatic patients.^[^ ^15^, ^16^, ^17^ ^]^ By modulating the size of NB, we can augment their penetration into the skin. The pH‐responsive coordination bonds of NB ensure the controlled release of BXP exclusively within the acidic microenvironment of the inflamed area, thereby sparing healthy tissues from unnecessary exposure. NB exhibited promising therapeutic efficacy in psoriasis, enhancing the bioavailability of BXP and mitigating its adverse effects.

In this study, we applied NB and further determined the possible downstream effectors of BXP. Using mass spectrometry to characterize nascent proteome after NB treatment in vitro, heat shock protein 90 alpha family class B member 1 (HSP90AB1) was identified to be significantly decreased in HEKa cell samples with NB. HSP90, belonging to heat shock proteins (HSP), can bind with the co‐chaperone cell division cycle 37 (CDC37) to recruit client proteins like STAT3 (signal transducer and activator of transcription 3), AKT (Protein Kinase B), JAK2 (Janus Kinase 2), *etc*.^[^ ^18^, ^19^, ^20^ ^]^ It was reported that HSP90 inhibitor was associated with clinically meaningful improvement in a subset of patients with plaque psoriasis.^[^ ^21^ ^]^ We further detected the efficacy of AUY922 (also known as Luminespib, NVP‐AUY922), a new generation of HSP90 inhibitor that was the most studied for a variety of cancers in Phase I and II clinical trials recently.^[^ ^22^ ^]^ Our study found that the therapeutic effect of NB treatment was superior to AUY922 in mice. NB not only decreased the expression of HSP90AB1 but the interaction with CDC37. Here, CUT&Tag was performed to investigate the possible transcription factors (TF) of HSP90AB1, and we reported that CCCTC‐binding factor (CTCF) transcriptionally activating HSP90AB1 and was downregulated by NB. In conclusion, our study prepared NB to exert anti‐psoriasis function through topical administration. The underlying mechanisms were investigated using in vitro and in vivo models and we demonstrated that NB could inhibit the transcription of HSP90AB1 via CTCF and disrupt the interactions of CDC37 leading to inactivation of the client STAT3. Hence, the present study provides valuable insights into the efficacy of nanomedicine, novel therapeutic targets and molecular mechanisms for psoriasis treatment.

## 2 Results

### 2.1 BXP Suppresses the Proliferation of HEKa Cells and Inhibits the Inflammation Response under M5 Treatment

**Figure 1:**
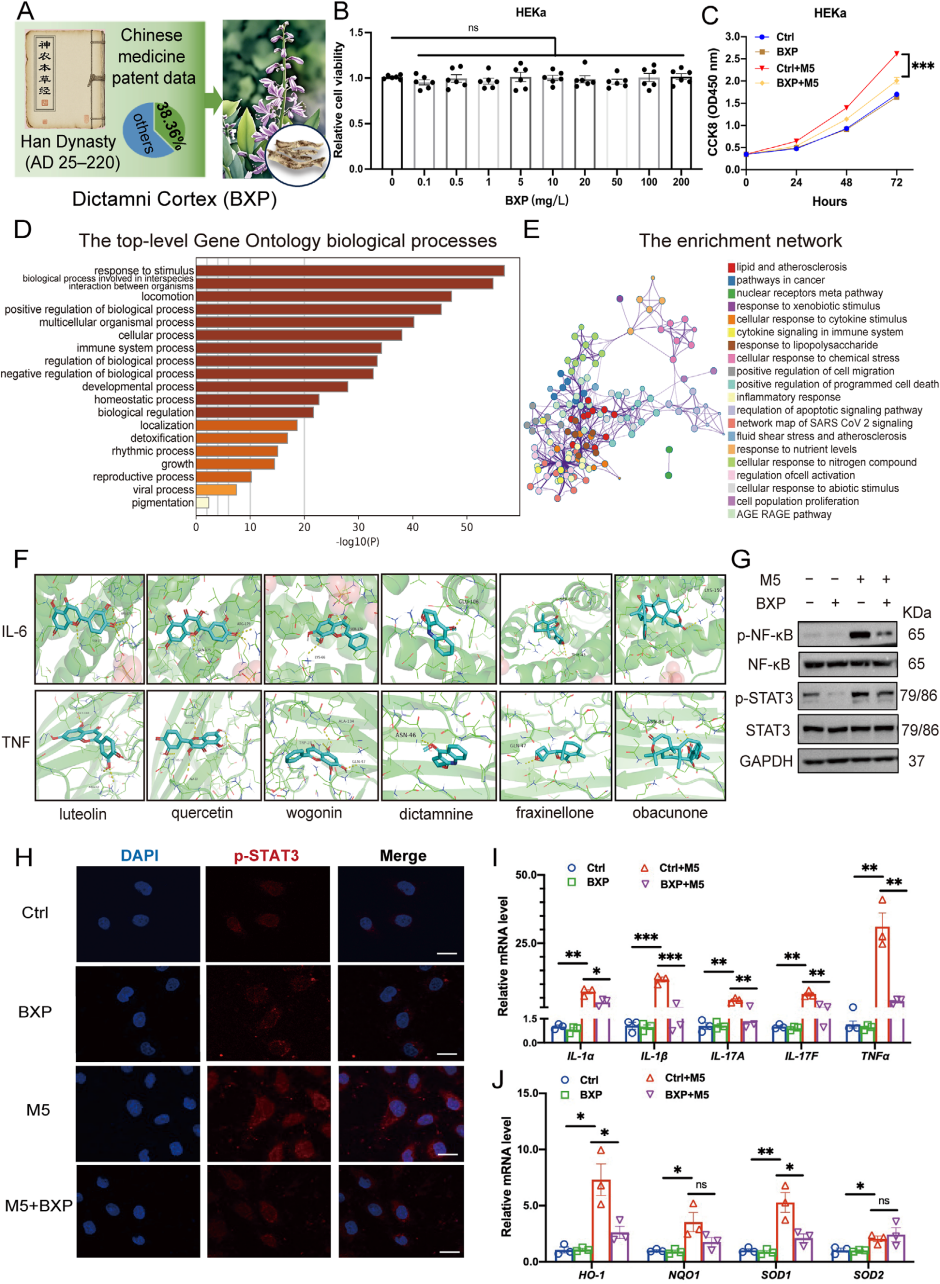
BXP Suppresses the Proliferation of HEKa Cells and Inhibits the Inflammation Response under M5 Treatment. A) The historical origin of BXP and the status of Chinese patent application. B) Cell viability of HEKa cells under treatment of different concentrations of BXP after 24 h. C) Detection of cell proliferation under M5 treatment and BXP (20 mg L^−1^) via CCK‐8 assay. D) The top‐level Gene Ontology biological processes and enriched network (E). F) Molecular docking of IL‐6 and TNF with luteolin, wogonin, quercetin, dictamnine, fraxinellone and obacunone. G) Immunoblotting of p‐NF‐κB/NF‐κB and p‐STAT3/STAT3 when cells were treated with or/and without M5 and BXP. H) Immunofluorescence of p‐STAT3 under treatment of BXP and M5 for 24 h. Scale bar = 10 µm. Relative mRNA levels of cytokines (IL‐1α, IL‐1β, IL‐17A, IL‐17F, and TNFα) I) and antioxidant system (HO‐1, NQO‐1, SOD1/2) J). Data are presented as mean ± SEM (*n* = 3 or 6 biologically independent cell samples). *p < 0.05, **p < 0.01, ***p < 0.001, ns, no significance, analyzed by two‐tailed Student's t test or two‐way ANOVA with Bonferroni's post hoc test.

BXP has been prescribed for skin diseases since ancient China. The first record of BXP was in Shennong's Herbal Classic of Materia Medica (神农本草经) and up to 38.36% of patent data of TCM compounds for the treatment of psoriasis used BXP (**Figure** **1**A). Multiple components of BXP were classified into five categories through LC‐MS/MS: flavonoids (luteolin, quercetin, wogonin, etc.), terpenoids (fraxinellone, obacunone, etc.), alkaloids (dictamnine, etc.), fatty acid derivatives and ceramides (Table S1, Supporting Information). The classification of major components was displayed in Figure S1 (Supporting Information). The whole spectrums of BXP and six active components (luteolin, quercetin, wogonin, dictamnine, fraxinellone and obacunone) along with the standard curves to calculate their contents in BXP, were displayed in Figures S2 and S3 and Table S2 (Supporting Information). In addition, the MS1 spectrums of representative fatty acid derivatives (palmitic acid, oleamide, stearamide) and ceramides (phytoceramide c2, c16 phytosphingosine, N,N‐dimethylsphingosine) were displayed in Figure S4 (Supporting Information). We previously found that different concentrations of BXP, varied from 0.1 – 200 mg L^−1^ had limited effect on HEKa cell viability in 24 h (Figure 1B) but concentrations over 50 mg L^−1^ could restrain the proliferation after 48 h (Figure S5A, Supporting Information). In M5‐induced psoriatic cell model, BXP (20 mg L^−1^) significantly suppressed abnormal keratinocyte proliferation, detected via CCK‐8 assay (Figure 1C). Given its anti‐inflammatory properties, we used molecular docking to identify potential targets of BXP. Not surprisingly, luteolin, quercetin and wogonin, three important components, were also among the compounds most frequently linked to the target genes of BXP. To verify possible targets of BXP, we performed molecular docking and the target lists of proteins from luteolin, quercetin, wogonin, dictamnine, fraxinellone and obacunone were imported to Metascape.^[^ ^23^ ^]^ The top‐level Gene Ontology biological processes were shown in Figure 1D as well as the most enriched network (Figure 1E). Notably, all six chemicals could bind to IL‐6 and TNF, underlying anti‐inflammation properties (Figure 1F). Thus, we further detected signaling transduction in inflammation and the results showed that BXP could inhibit the phosphorylation of STAT3 and NF‐κB under M5 treatment (Figure 1G, quantification results in Figure S5B,C, Supporting Information).

Immunofluorescence also displayed the same results that fluorescence intensity of p‐STAT3 was significantly decreased with BXP treatment (Figure 1H). Moreover, mRNA levels of cytokines (IL‐1α, IL‐1β, IL‐17A, IL‐17F, and TNFα) were significantly downregulated (Figure 1I). In addition, BXP (as a natural extract) exerts therapeutic effects by inhibiting oxidative stress. M5 treatment disrupted the oxidative balance, as evidenced by upregulated HO‐1, NQO‐1 and SOD1/2 expression. However, BXP significantly curbed the elevated expression levels of HO‐1 and SOD1 (Figure 1J).

### 2.2 Preparation and Characterization of NB

**Figure 2:**
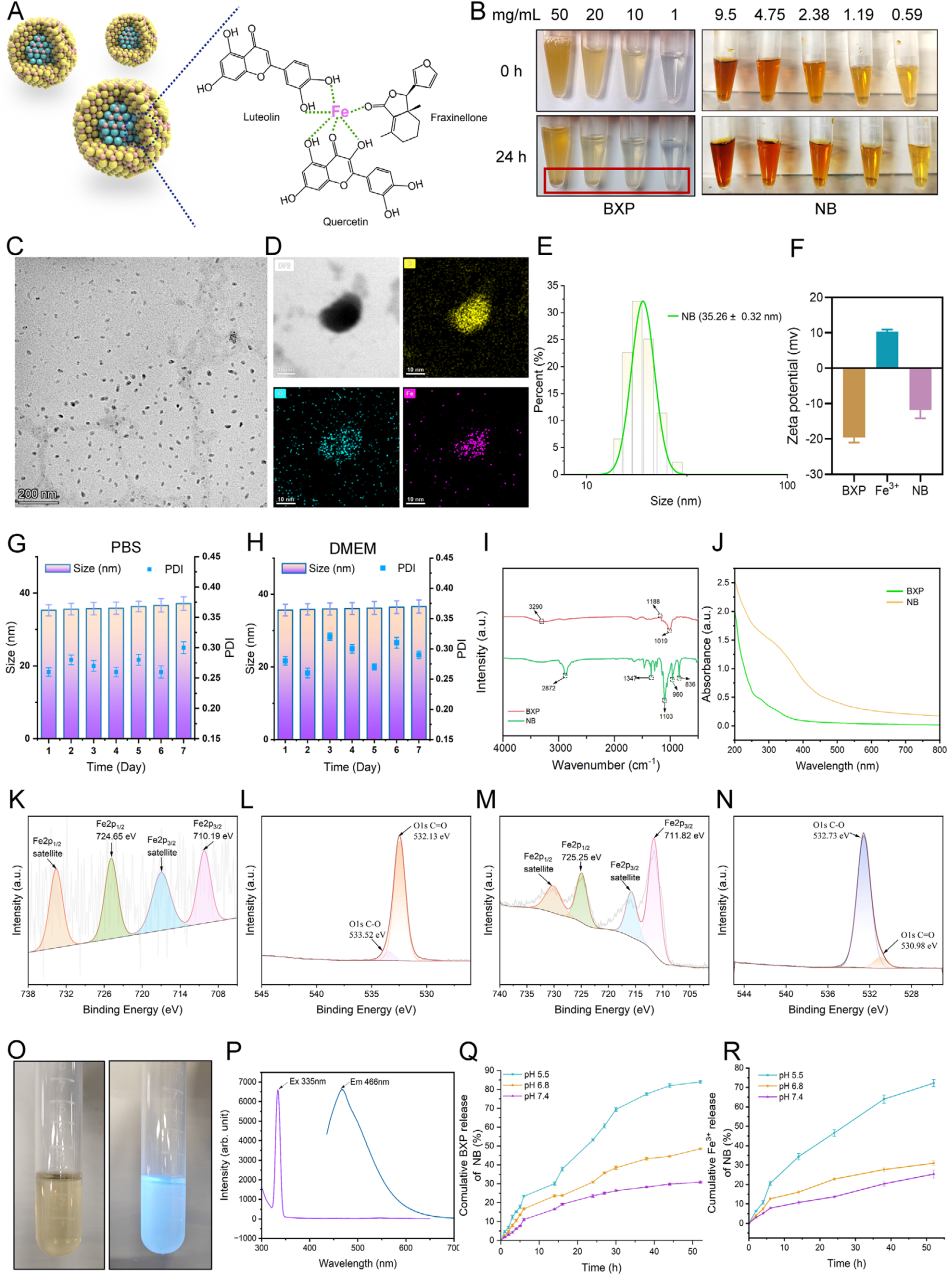
Preparation and characterization of NB. A) The sketch of NB construction. B) Poor water solubility of BXP with different concentrations compared with NB. C) TEM plot of NB particles. D) Lorenz TEM plot (scale bar = 100 nm) and energy spectrum (EDS) of NB particles. E,F. Hydrated particle size (E) and Zeta potential (F) of NB. G,H) Changes in particle size of NB in PBS solution (G) and medium (H) over 7 days. I) Fourier transform infrared (FTIR) spectra of NB. J) The UV–vis absorption spectra of NB. K,L) X‐ray photoelectron spectra (XPS) of NB, including Fe 2p spectra (K) and O 1s spectra (L). M) Fe 2p spectra of FeCl_3_. N) O 1s spectra of BXP. O,P) Picture (O) and fluorescence spectrum (P) of autofluorescence of NB excited by ultraviolet light. Q‐R) The amount of BXP (Q) and Fe^3+^ (R) released by NB particles at different time points under different pH environments (pH = 5.5, 6.8, and 7.4). Data in F‐H are presented as mean ± SEM (*n* = 3 biologically independent samples).

While topical and oral applications of TCM are effective and convenient for psoriasis patients, transcutaneous delivery remains challenging due to the poor water solubility and low bioavailability of TCM.^[^ ^24^ ^]^ BXP was not an exception. Here, we constructed BXP‐Fe (III) ICPs (NB) to overcome the shortcomings as mentioned. HPLC‐MS analysis of BXP shows it contains diverse natural products, mainly flavonoids, alkaloids, terpenoids, and ceramides. The ketone, phenolic hydroxyl, and imino groups in these compounds can link via coordination bonds around each Fe^3+^ center. This forms a 3D structure that expands outward, self‐assembling into supramolecular infinite coordination polymer nanoparticles. Focusing on six BXP compounds key for psoriasis treatment, we illustrate the possible coordination between BXP and Fe^3+^ to form NB (**Figure** **2**A; Figure S6A, Supporting Information). Due to the differential coordination affinities of the individual constituents toward Fe^3+^, NB may contain Fe^3+^ centers coordinated by one or two ligands, or by multiple ligands in a random fashion. Only the potentially existing coordination structures are presented. Notably, epidermal iron has been reported significant decrease in the epidermis of psoriatic patients.^[^ ^15^, ^16^, ^17^ ^]^ Nanoparticles with different BXP to Fe^3+^ weight ratios (1:1, 1:2, 1:3, 1:4) were synthesized to optimize nanoproperties. At m(BXP): m(Fe^3+^) = 1:1, uniform nanoparticles weren't obtained, likely due to insufficient Fe^3+^ for coordination with BXP. At m(BXP): m(Fe^3+^) = 1:4, nanoparticle formation failed as excessive Fe^3+^ caused violent coordination and excessive growth, leading to aggregation. Uniform nanoparticles were achieved at m(BXP): m(Fe^3+^) = 1:2 or 1:3, but the 1:2 ratio provided the best long‐term stability. Thus, we selected the BXP to Fe^3+^ mass ratio of 1:2 for subsequent research. BXP precipitated within 24 h at all tested concentrations (50, 20, 10, 1 mg mL^−1^ in water). In contrast, NB remains soluble at concentrations of 9.5, 4.75, 2.38, 1.19, and 0.59 mg mL^−1^ (Figure 2B). The maximum aqueous solubility of BXP is ≈0.1 mg mL^−1^ (Figure S6B, Supporting Information). Thus, NB enhanced the aqueous solubility of BXP by 95‐fold. Transmission electron microscopy (TEM) and dynamic light scattering (DLS) results showed that NB were in the shape of a broad bean with a hydrodynamic diameter of 35.26 ± 0.32 nm (Figure 2C–E). DLS further demonstrated that the zeta potentials of BXP and Fe^3+^ were −19.5 and 10.3 mV, respectively, while NB showed −11.8 mV after positive ions were coordinated with BXP (Figure 2F). The loading amount is 94.09 ± 2.76%, as determined by fluorescence spectrophotometer. Furthermore, the results of Lorenz TEM and energy spectroscopy (EDS) demonstrated that carbon (C), oxygen (O) and iron (Fe) were distributed uniformly within NB. The particles size of NB changes very little over 7 days in PBS (pH 7.4) (Figure 2G) and DMEM medium (Figure 2H), ensuring their long‐term stability. Fourier transform infrared spectroscopy (FT‐IR) confirms the BXP in NB, as shown by the peaks at 3290 and 1019 cm^−1^ (Figure 2I). For instance, the peak at 2883 cm^−1^ shows a significant weakening of the O‐H stretching vibration due to the replacement of the proton by Fe^3+^ and the formation of a new, stronger hydrogen‐bonding peak at 2872 cm^−1^, whereas the rearranging of the electron cloud density and the enhancement of the polarity after the coordination of the C‐O bond with Fe^3+^ at 1019 cm^−1^ lead to an increase in the intensity of the peaks and an increase in the vibrational frequency. The UV–vis absorption spectra reconfirm the presence of BXP in NB (Figure 2J).

X‐ray photoelectron spectroscopy (XPS) was used to elucidate the coordination mechanism of BXP and Fe^3+^. The XPS survey spectrum of BXP and NB as well as high‐resolution XPS spectra of C 1s were presented in Figure S6C–F (Supporting Information). The high‐resolution Fe 2p spectra of FeCl_3_ shows Fe 2p_3/2_ and Fe 2p_1/2_ at 711.82 and 725.25 eV (Figure 2M). The high‐resolution Fe 2p spectra showed Fe 2p_3/2_ and Fe 2p_1/2_ at 710.19 and 724.65 eV in NB, respectively, which indicated the presence of Fe as Fe^3+^ in NB. The high‐resolution O 1S spectra of BXP showed characteristic peaks at 530.98 and 532.73 eV, while the high‐resolution O 1S spectra of NB showed characteristic peaks at 533.52 and 532.13 eV. This suggests that Fe^3+^ is coordinated to BXP by sharing the electron pair of O atoms, and the electron donation effect of O atoms to Fe^3+^ ions leads to higher binding energies for O 1s and lower binding energies for Fe 2p (Figure 2K–N). Surprisingly, we found that BXP could display autofluorescence after the excitation of ultraviolet light (Figure 2O) and measured the maximum excitation wavelength (Ex) at 405 nm and the maximum emission wavelength (Em) at 468 nm of BXP by fluorescence spectrophotometry (Figure 2P). We speculate that BXP might contain compounds with intrinsic fluorescence, such as quercetin. Coordination bonds are formed by atoms with lone pairs of electrons (ligands) and atoms with empty orbitals (metal ions). pH changes can alter the properties of ligands and central ions, causing ligand protonation or deprotonation. Moreover, Fe^3+^ forms different hydrated ions under varying pH conditions. It was reported that psoriasis patients exhibited elevated levels of circulating amino acids and metabolites from the glycolytic pathway, lactate accumulation in the skin or blood leading to the formation of an acidic environment, which was predisposed to aggravate inflammation of psoriasis.^[^ ^25^, ^26^, ^27^, ^28^ ^]^ NB exhibits pH‐responsive drug release, which makes it suitable for the acidic microenvironment of psoriatic lesions. We found that under physiological conditions (pH 7.4), less than 30% of BXP was released from NB within 52 h, indicating that the nanocomposites remain stable in neutral pH environment (Figure 2Q). In addition, the release rate of BXP was significantly increased under more acidic conditions (pH 5.5), with more than 80% of BXP released within 52 h, confirming the pH responsiveness of the NB (Figure 2Q). In addition, inductively coupled plasma mass spectrometry (ICP‐MS) measurements of the release profile of Fe^3⁺^ from NB showed that the release trend of Fe^3+^ was closely related to BXP (Figure 2R). In psoriatic lesion area, lactate was accumulated to supply an acidic environment. The augmented release of BXP and Fe^3+^ under pH 5.5 rather than pH 6.8 and pH 7.4 indicated the targeted delivery capability and anti‐inflammation properties of NB.

### 2.3 NB Inhibits the Abnormal Proliferation and Inflammatory Response in Cells

**Figure 3:**
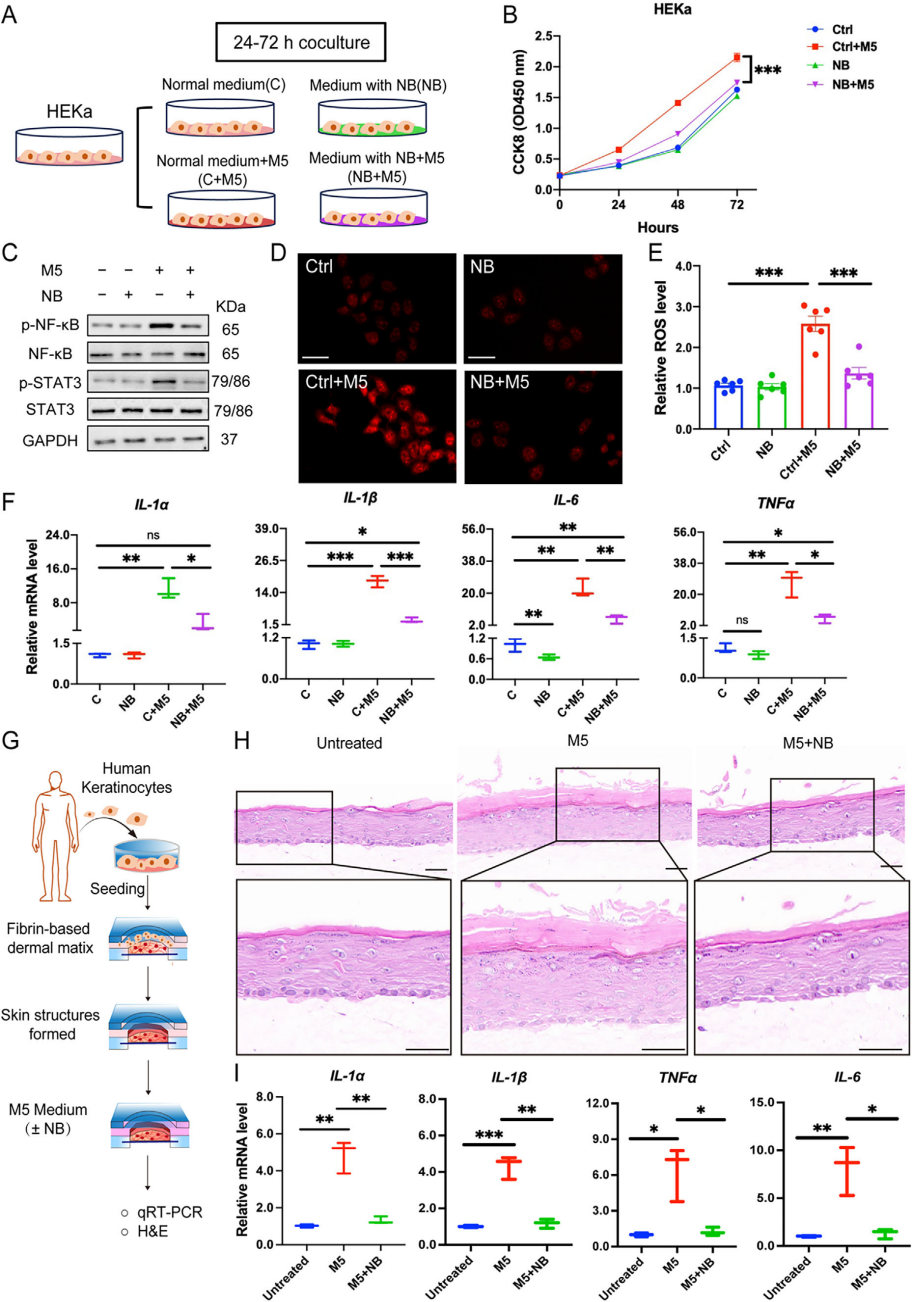
NB inhibits the abnormal Proliferation and inflammatory response in cells. A) The flow chart of M5 and NB treatment in HEKa cells. B) Quantification of cell proliferation under different treatments at 24, 48, and 72 h via CCK‐8 assay. C) Immunoblotting of p‐NF‐κB/NF‐κB and p‐STAT3/STAT3 when cells were treated with or/and without M5 and NB. D) DHE staining of cells reflecting ROS level. E) ROS level measurement via DCFH‐DA. F) Relative mRNA levels of cytokines (IL‐1α, IL‐1β, IL‐6, and TNFα). G) The sketch of NB and M5 in microfluidic skin organ‐on‐a‐chip. H) H&E staining of three groups. I) Relative mRNA levels of cytokines (IL‐1α, IL‐1β, IL‐6, and TNFα). Data are presented as mean ± SEM (*n* = 3 or 6 biologically independent cell samples). *p < 0.05, **p < 0.01, ***p < 0.001, ns, no significance, analyzed by two‐tailed Student's t test or two‐way ANOVA with Bonferroni's post hoc test.

We previously found that different concentrations of BXP, varied from 0.1 – 200 mg L^−1^ had limited effect on HEKa cell viability in 24 h. When cells were treated with M5 (a cocktail of cytokines, including TNF‐α, IL‐17A, IL‐22, IL‐1α, and Oncostatin‐M, 10 ng mL^−1^), as the flow chart shown in **Figure** **3**A, both BXP and NB (20 mg L^−1^) could suppress the excess proliferation of cells (Figure 3B). Considering BXP is a mixture of multiple pharmacological components, we examined the anti‐proliferation of individual monomers (luteolin, quercetin, wogonin, fraxinellone, obacunone and dictamnine), cross‐category combinations (flavonoid, terpenoid, alkaloid), and a complete mixture of all compounds (MIX) with their natural abundance in BXP using the CCK‐8 assay (Figure S7A–C, Supporting Information). Immunoblotting results showed that the inhibition ability of STAT3 phosphorylation closely mirrored the CCK‐8 results (Figure S7D, Supporting Information). Notably, individual compounds, their combinations, or the physical mixture all failed to achieve comparable efficacy to BXP or NB, underscoring that the intrinsic synergistic activity among the herbal components cannot be replicated by single agents or simple physical mixing.

In addition, NB also showed the anti‐inflammatory effect, capable of inhibiting the phosphorylation of STAT3 and NF‐κB (Figure 3C), quantification results in Figure S8A,B, Supporting Information). Consisting with results of BXP, antioxidant ability was also noticed in NB (Figure 3D,E). As expected, NB treatment obviously blocked expressions of cytokines (IL‐1α, IL‐1β, IL‐6 and TNFα) (Figure 3F). We further detected the therapeutic efficacy and safety of NB in microfluidic skin organ‐on‐a‐chip (epidermal inoculation with human keratinocytes and dermal embedding in fibroblast/collagen matrix) (Figure 3G). H & E staining demonstrated that NB could successfully resist M5‐induced hyperproliferation of keratinocytes and overdosed cytokines (Figure 3H,I).

### 2.4 Subcutaneous Administration of NB Protects Mice Against Psoriatic Skin

Next, based on above data, we evaluated the therapeutic efficacy of NB via subcutaneous administration in IMQ‐induced psoriasis mice model. It has been reported that intragastric administration of high dose of BXP (over 0.9 g kg^−1^) can induce hepatotoxicity.^[^ ^29^ ^]^ Subcutaneous administration of NB could improve targeting and stability, reduce blood intake and organ accumulation. Here, we chose 20 mg kg^−1^ referred in a study of BXP on atopic dermatitis^[^ ^30^ ^]^ and based on our pre‐experiments. We found a dose‐dependent efficacy in NB that protective ability of 10 mg kg^−1^ treatment was weaker than 20 and 50 mg kg^−1^ NB (Figure S9A,B, Supporting Information). However, 50 mg kg^−1^ NB could cause partly decrease in epidermal moisture content after continuous injection (Figure S9C,D, Supporting Information). 20 mg kg^−1^ NB would not influence organ functions after 14‐day injection, since H&E staining revealed that the structures of heart, liver, lung, kidney and spleen remained normal with no typical features of cell necrosis or inflammatory infiltrates observed (Figure S9E, Supporting Information). Creatinine (CR), aspartate aminotransferase (AST) and alanine aminotransferase (ALT) levels were checked to assess potential side effects of NB. When compared with untreated group, these indicators remained stable, indicating normal function of liver and kidney (Figure S9F, Supporting Information). Taken together, 20 mg kg^−1^ NB was applied in the following experiments.

**Figure 4:**
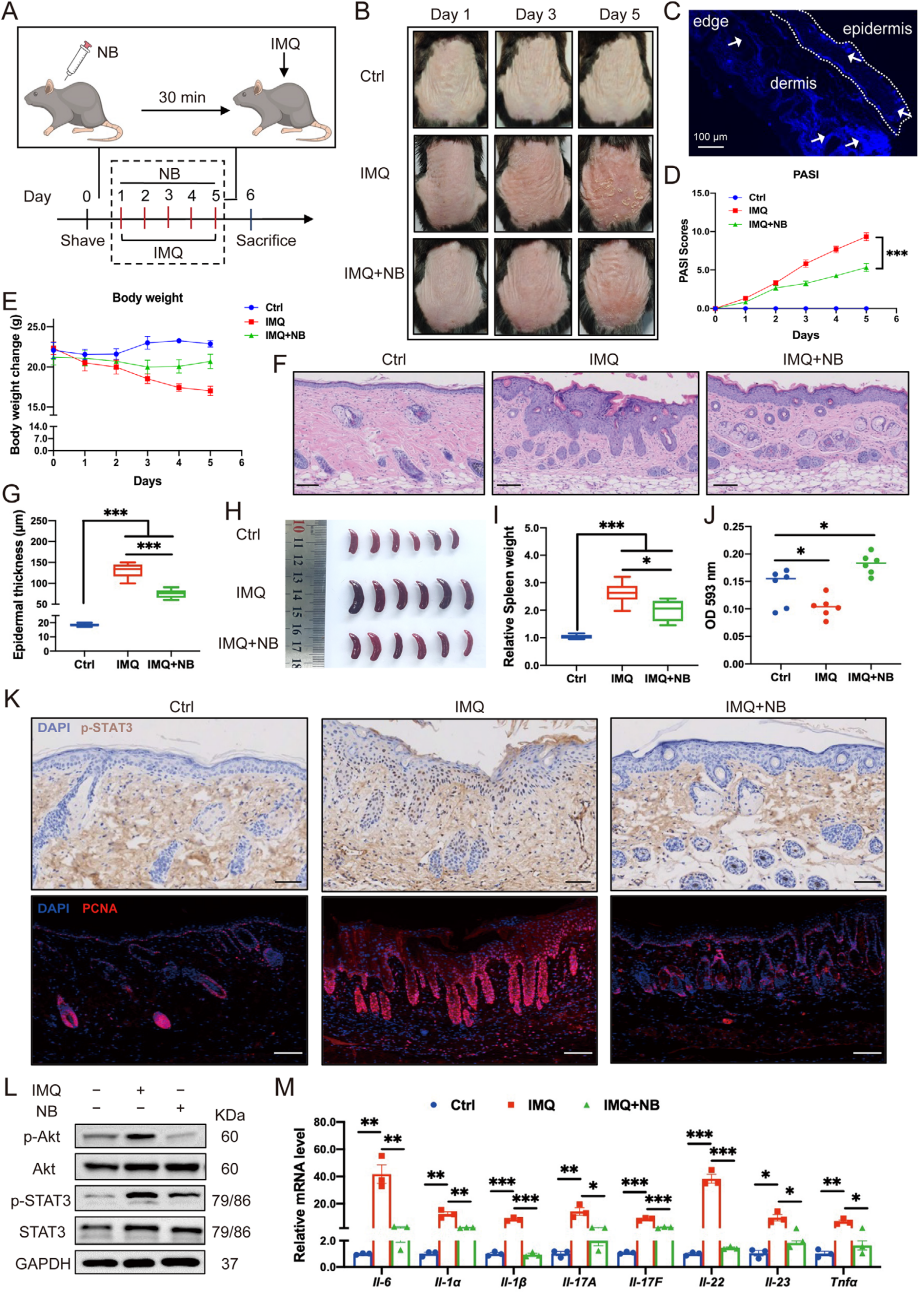
Subcutaneous administration of NB protects mice against psoriatic skin. A) The sketch of the whole experiment procedure. B) Representative images of three groups of mice on day 0, day 3, and day 5. C) Detection picture of skin tissue sections from NB treatment group by fluorescence microscopy. The arrows pointed to significant fluorescence aggregation. D) PASI scores of each mouse in three groups. E) Body weight changes of the mice during the treatments. F) H&E staining of the back skin of the mice from different groups. G) Thickness of the epidermis was measured according to H&E results. H) Spleen images and relative spleen weight change to body weight (I) (normalized to the control group). J) Iron content was quantified at OD 593 nm by Total Iron Colorimetric Assay Kit. K) Immunohistochemistry of p‐STAT3 and immunofluorescence of PCNA of skin sections. L) Immunoblotting of p‐Akt/Akt and p‐STAT3/STAT3 of mice skin tissues from different groups. M) Relative mRNA levels of psoriasis‐associated cytokines (*Il‐6*, *Il‐1α*, *Il‐1β*, *Il‐17A*, *Il‐17F*, *Il‐22*, *Il‐23*, *Tnfα*). Scale bar = 100 µm. Data are presented as mean ± SEM (*n* = 3 or 6 mice per group). *p < 0.05, **p < 0.01, ***p < 0.001, ns, no significance, analyzed by two‐tailed Student's t test or two‐way ANOVA with Bonferroni's post hoc test.

To examine in vivo functions of NB, C57BL/6J mice were randomly divided into three different groups: Ctrl, IMQ and IMQ+NB (*n* = 6 per group). The sketch of the whole procedure was shown in **Figure** **4**A. After shaving, mice in IMQ+NB group were subcutaneously administered with NB (20 mg kg^−1^) 30 min prior to topical application of 5% IMQ for continuous 5 days. Photos of the back skin were captured every day and NB protected mice from IMQ‐induced psoriatic skin (Figure 4B). With the self‐luminous ability of BXP, we found that the skin sections could appear fluorescent after the administration of NB, underlying increased bioavailability (Figure 4C, arrows pointed to the deposit area). In addition, NB significantly ameliorated IMQ‐induced psoriasis in mice, decreasing the clinical score of the psoriasis area and severity index (PASI) (Figure 4D), protecting the mice from weight loss caused by IMQ (Figure 4E), suppressing epidermal hyperplasia and splenomegaly (Figure 4F–I). In addition, as NB was a coordination polymer containing ions, we detected iron content from skin samples via Total Iron Colorimetric Assay Kit and found that NB increased iron content compared with IMQ group (Figure 4J; Figure S9G, Supporting Information). For the anti‐inflammatory and anti‐proliferation property, lower levels of p‐STAT3, p‐Akt and PCNA were observed in IMQ+NB group (Figure 4K,L, quantification results in Figure S9H,I, Supporting Information) as well as mRNA levels of psoriasis‐associated cytokines (*Il‐6*, *Il‐1α*, *Il‐1β*, *Il‐17A*, *Il‐17F*, *Il‐22*, *Il‐23*, *Tnfα*) (Figure 4M).

### 2.5 Nascent Proteomics Changes Triggered by NB Determines HSP90AB1 as the Target

**Figure 5:**
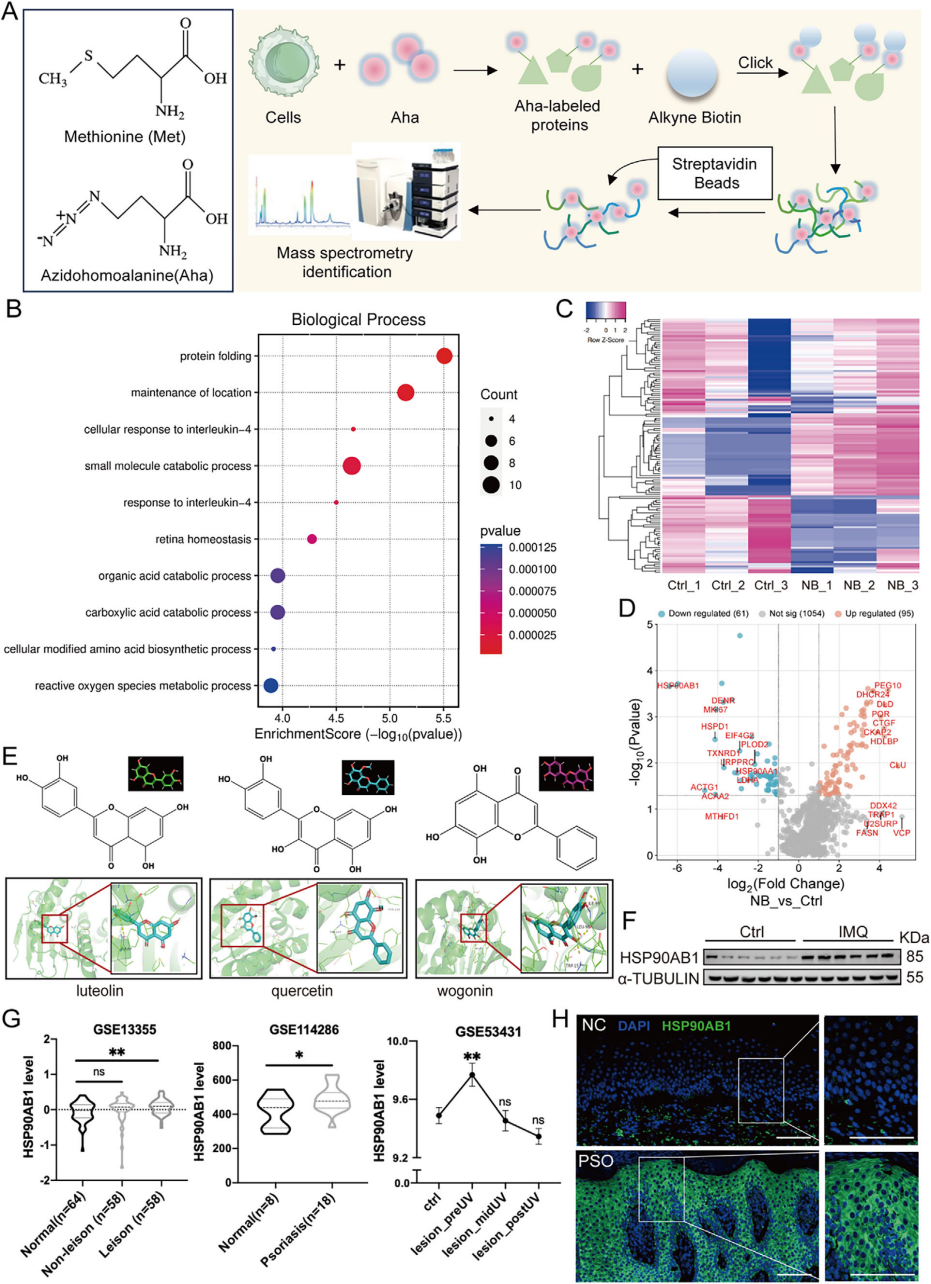
Nascent proteomics changes triggered by NB determines HSP90AB1 as the target. A) A schematic diagram of Aha‐labeled nascent proteome detection. B) Several main enriched biological processes. C) The heatmap and (D) volcano plot of significantly changed nascent proteins upon NB treatment. E) Molecular docking of HSP90AB1 and luteolin, wogonin and quercetin. F) Immunoblotting of HSP90AB1 in IMQ‐stimulated mice skin versus control group. G) HSP90AB1 expression level sequenced from GEO database (GSE13355, GSE114286, and GSE53431). H) Immunofluorescence of HSP90AB1 of skin sections from healthy donates (*n* = 3) and psoriasis patients (*n* = 3), scale bar = 100 µm. Data are presented as mean ± SEM (*n* = 3 biologically independent cell samples or 6 mice per group). The cut‐off of MS was set as |log_2_ fold change| ≥ 1 and *p*‐value < 0.05. *p < 0.05, **p < 0.01, ***p < 0.001, ns, no significance, analyzed by two‐tailed Student's t test or two‐way ANOVA with Bonferroni's post hoc test.

To investigate the possible target of NB, we utilized azidohomoalanine (Aha) known as the analog of methionine that we have described before,^[^ ^31^, ^32^, ^33^ ^]^ to pulse newly synthesized proteins after NB treatment in HEKa cells, which might be the ones directly influenced by NB. After culturing with Aha to substitute methionine, nascent proteins were labeled then coupled with alkynl‐biotin through click chemistry reaction, followed by enrichment with streptavidin beads and mass spectrum quantification for further detection (**Figure** **5**A). Enrichment of the pathway from NB versus Ctrl. including ferroptosis underlying Fe^3+^ functions of NB (Figure S10A,B, Supporting Information). Several main enriched biological processes including protein folding, maintenance of location, cellular response to interleukin‐4 and small molecule catabolic process were listed in Figure 5B. GO results of three ontologies are shown in Figure S10C (Supporting Information).The significant changes in nascent proteins upon NB treatment were shown in the heatmap^[^ ^34^ ^]^ and volcano plot (Figure 5C,D). Among the 156 significantly altered proteins in the nascent proteome, HSP90AB1 was identified due to its pronounced downregulation following NB intervention. In addition, HSP90AB1 could also be recognized as a common target for luteolin, wogonin, quercetin and obacunone from BXP (Figure 5E; Figure S10D, Supporting Information). Next, we evaluated protein and mRNA level of HSP90AB1 in IMQ‐stimulated mice skin and found an increase (Figure 5F, quantification results in Figure S10E,F, Supporting Information). Sequencing data from GEO database (GSE13355, GSE114286, and GSE53431) revealed that HSP90AB1 expression was notably increased in lesion skin from psoriasis patients compared to the normal donates and UV treatment could lower the expression (Figure 5G). Based on the above results, we collected skin sections from psoriasis patients and found HSP90AB1 was markedly higher in the epidermal when compared with the normal population (Figure 5H). Skin sections were obtained from the tissue bank of the department of dermatology at the First Affiliated Hospital of Xi'an Jiaotong University with the informed consent waiver and approved by the Ethics Committee of Xi'an Jiaotong University (XJTU1AF2025LSYY‐329).

### 2.6 HSP90 Inhibitor AUY922 Moderately Ameliorates IMQ‐Induced Psoriasis in Mice

**Figure 6:**
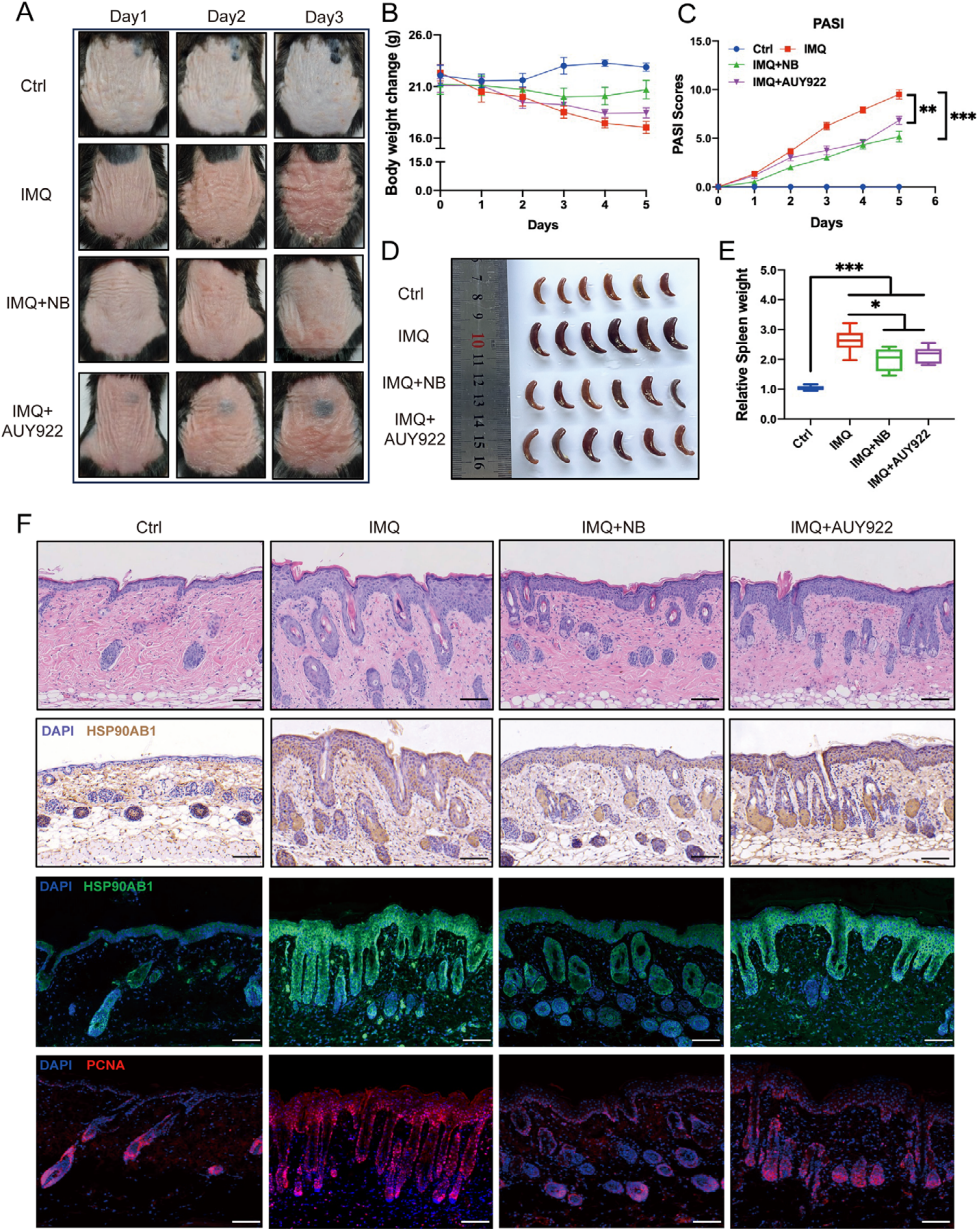
HSP90 inhibitor AUY922 moderately ameliorates IMQ‐induced psoriasis in mice. A) Representative images of four groups of mice on day 0, day 3, and day 5. B) Body weight change and (C) PASI of the mice from four groups. D) Spleen images and relative spleen weight change to body weight (E) (normalized to the control group). F) H&E staining and immunohistochemistry of HSP90AB1 as well as immunofluorescence of HSP90AB1 and PCNA of skin sections from four groups. Data are presented as mean ± SEM (*n* = 6 mice per group). *p< 0.05, **p< 0.01, ***p < 0.001, ns, no significance, analyzed by two‐tailed Student's t test.

The previous results hinted that HSP90AB1 might be an important molecular functioning in the development of psoriasis. Herein, we applied AUY922, a well‐known N‐terminal HSP90 inhibitor (with IC_50_ values of 7.8 and 21 nM for HSP90α and HSP90β) for its anti‐tumor property in IMQ‐induced psoriasis mouse model. We realized that subcutaneous application of AUY922 could ameliorate psoriatic syndromes to some extent with lower PASI, weight loss and suppressed splenomegaly (**Figure** **6**A–E). Furthermore, skin sections were collected for H&E, immunohistochemical staining (HSP90AB1 antibody) and immunofluorescence (HSP90AB1 and PCNA antibody). Comparing with NB group, AUY922 group displayed faintish functions to counter IMQ stimulation (Figure 6F). Taken together, the therapeutic efficacy of AUY922 was inferior to NB. Next, we dug deeper that how NB could influence the expression level and functions of HSP90AB1.

### 2.7 NB Disrupts the Binding of the Co‐Chaperone CDC37 with HSP90AB1 to Reduce Biological Transductions of the Clients

**Figure 7:**
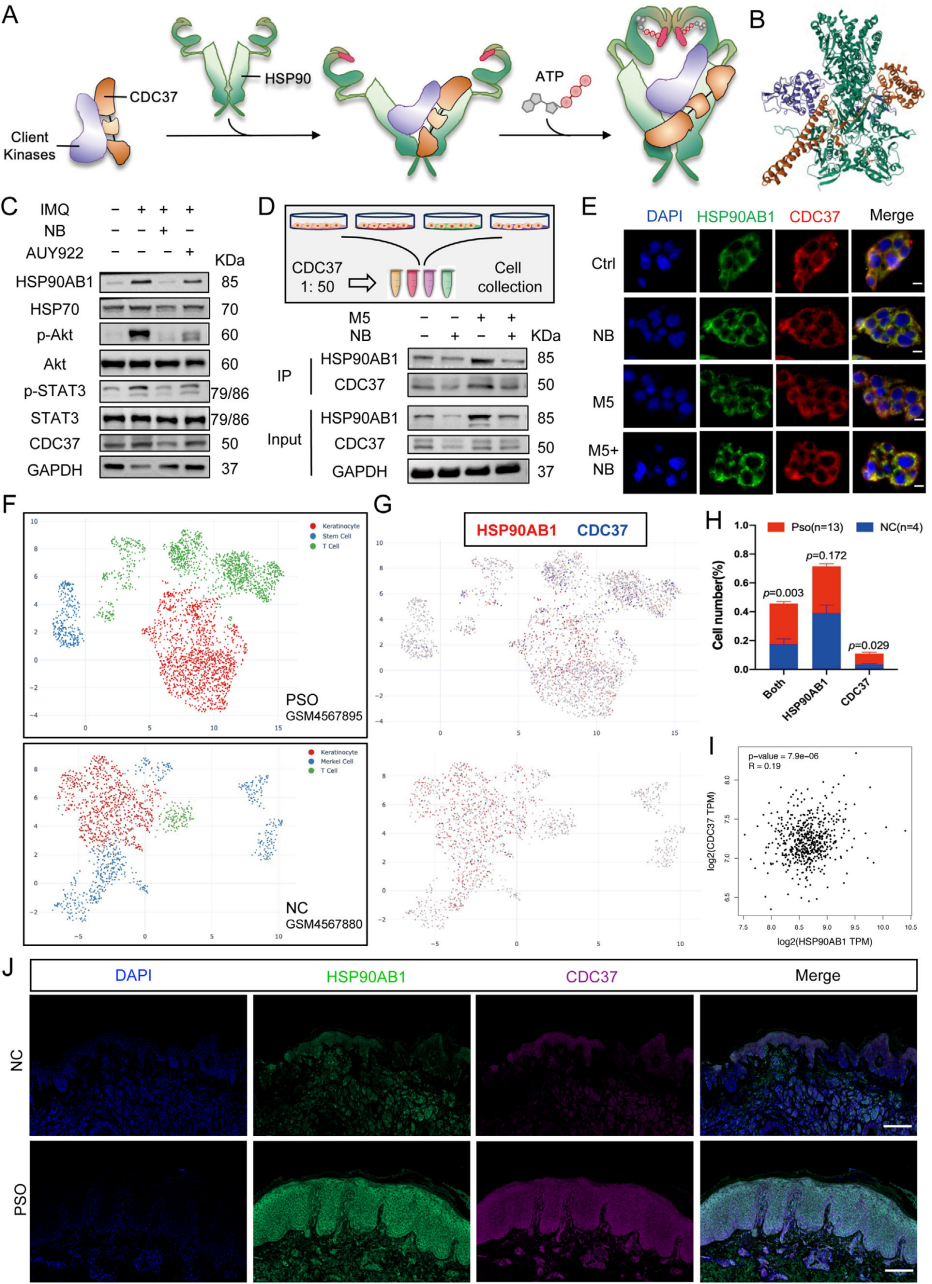
NB disrupts the binding of the co‐chaperone CDC37 with HSP90AB1 to reduce biological transductions of the clients. A) The binding procedure of the co‐chaperone and the client kinases. B) CDC37 (Orange) and CDK4 (Purple) bound to HSP90β (Green) (PDB ID: 5FWL). C) Immunoblotting of HSP90AB1, HSP70, p‐Akt/Akt, p‐STAT3/STAT3 and CDC37 of the skin samples from four groups of mice. D) CO‐IP of HSP90AB1 and CDC37 in HEKa cells with/without NB and M5 treatment. E) Immunofluorescence of HSP90AB1 and CDC37 in HEKa cells, scale bar = 20 µm. F) Single‐cell RNA sequencing of emigrating cells from human psoriasis skin and control normal skin (Dataset ID: SCDS0000417): patient (GSM4567895) and normal control (GSM4567880). G) Gene expressions of HSP90AB1 and CDC37 from samples in (F). H) Statistical analysis of positive cell numbers of HSP90AB1 and CDC37 in Pso (*n* = 13) and NC (*n* = 4) from the dataset. I) Correlation analysis (Pearson) of HSP90AB1 and CDC37 basing on GEPIA‐GTEx. J) Immunofluorescence of HSP90AB1 and CDC37 in patients’ sections and normal controls. Scale bar = 200 µm. Data are presented as mean ± SEM (*n* = 3 biologically independent samples). *p < 0.05, **p < 0.01, ***p < 0.001, ns, no significance, analyzed by two‐tailed Student's t test.

Functions of HSP family, are influenced by many co‐chaperones which could coordinate the interplay between HSP90 and other chaperone systems, such as HSP70; stimulate/inhibit ATPase activity of HSP90; or recruit clients and other co‐chaperones for the biological processes.^[^ ^35^ ^]^ CDC37 is one of the best‐studied clients‐specific co‐chaperones of HSP90, and their clients almost exclusively associated with protein kinases, such as AKT and JAK1/JAK2, as well as transcription factors STAT3, *etc*.,^[^ ^18^, ^19^, ^20^ ^]^ which could drive keratinocyte hyperproliferation. A simplified binding sketch of CDC37 to HSP90 was depicted in **Figure** **7**A. The modular structure of the complex displayed an extensive contact with the kinase and HSP90 (Figure 7B). We then detected relative protein levels of HSP90AB1, HSP70, p‐Akt/Akt, p‐STAT3/STAT3, and CDC37 of the skin samples from four groups of mice (Figure 7C, quantification results in Figure S11, Supporting Information). Overall, AUY922 did not change the expression level of HSP90AB1 other than NB, which induced prominent decrease after the administration. Consistently, IMQ treatment augmented HSP90AB1 and activated signaling pathways, however, NB treatment not only inhibited Akt and STAT3 signaling but also reduced protein level of HSP90AB1 and CDC37. As for the inhibitor, AUY922, aiming at N‐terminal of HSP90, could hardly change the protein level of the co‐chaperone. We then examined the interaction of HSP90AB1 and CDC37 in HEKa cells and the co‐IP results demonstrated that their binding was weakened under NB treatment (Figure 7D). Moreover, immunofluorescence of HSP90AB1 and CDC37 displayed similar results that the colocalization was significantly decreased when cells were treated with NB neglecting M5 stimulation (Figure 7E). Next, we sought to verify the status of the co‐chaperone in psoriasis patients. Single‐cell RNA sequencing of emigrating cells from human psoriasis skin and control normal skin (Dataset ID: SCDS0000417) were applied for the follow‐up analysis. Cell information of a patient (GSM4567895) and normal control (GSM4567880) was shown in Figure 7F representatively. Gene expressions of HSP90AB1 and CDC37 were further revealed in Figure 7G. Here, we analyzed positive cell numbers of HSP90AB1 and CDC37 from 13 psoriasis patients and 4 negative controls. Interestingly, though there was no significance in HSP90AB1 positive cell numbers between Pso and NC, CDC37 positive and both positive cells were increased in the patients (Figure 7H). Correlation analysis (Pearson) of HSP90AB1 and CDC37 basing on GEPIA^[^ ^36^ ^]^‐GTEx (Skin‐Not Sun Exposed and Skin‐Sun Exposed) reflected high coefficient (Figure 7I).

Moreover, immunofluorescence of HSP90AB1 and CDC37 in psoriasis patients’ sections displayed higher intensity and colocalization when compared with the normal population (Figure 7J). Thus, we hypothesized that the HSP90AB1 and co‐chaperone CDC37 complex might be a novel target in future psoriasis treatment.

### 2.8 NB Influences the Expression Level of HSP90AB1 through Transcription Factor

**Figure 8:**
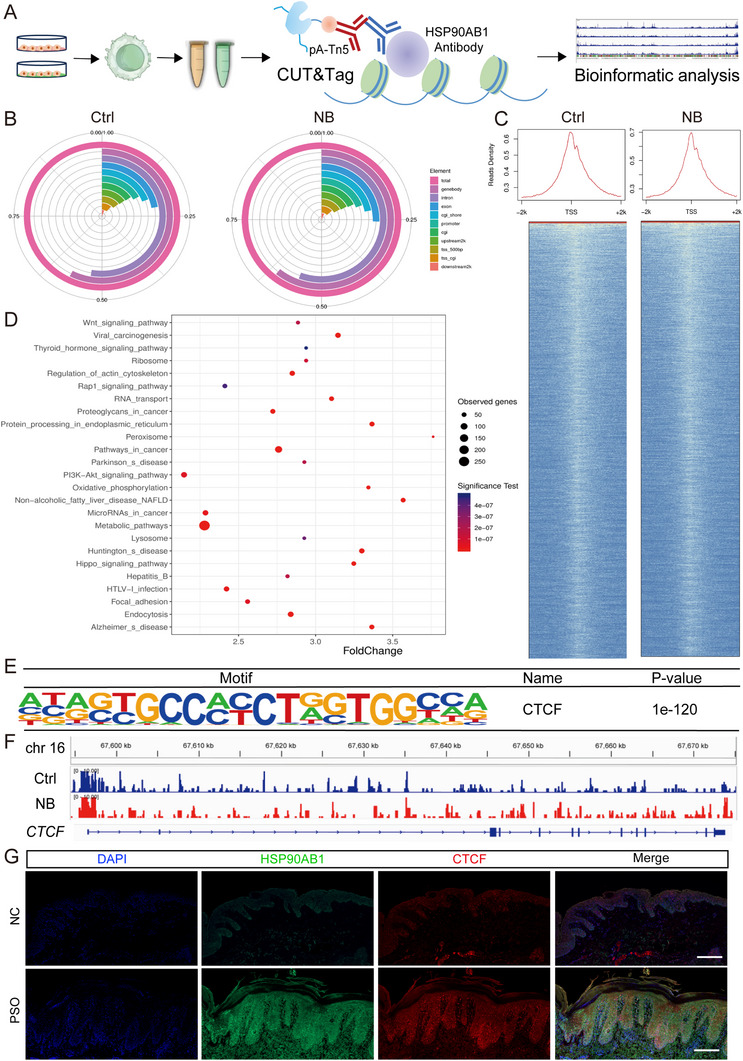
NB influences the expression level of HSP90AB1 through transcription factor. A) The flow chart of CUT&Tag. B) Enrichment peak annotated to the distribution of functional components from NB and Control group. C) The transcriptional start site (TSS) of NB and Control group. D) The most enriched pathways in NB versus Control. E) Homer known motif of CTCF. F) IGV visual analysis of CTCF in NB and Control group. G) Immunofluorescence of HSP90AB1 and CTCF in patients’ sections (*n* = 3) and normal controls (*n* = 3). Scale bar = 200 µm. Data are presented as mean ± SEM (*n* = 3 biologically independent cell samples).

In view of previous results, we aimed to figure out how the expression of HSP90AB1 could be affected upon NB treatment. We hypothesized that the transcription of *HSP90AB1* might be inhibited, thus, it was worth investigating that which transcription factor exactly worked. Here, we first utilized online tool Cistrome Data Browser^[^ ^37^, ^38^ ^]^ to predict possible transcription factors of HSP90AB1 and their Pearson's correlations (Figure S12A,B, Supporting Information). To identify the key transcription factors, we further performed CUT&Tag as described in **Figure** **8**A. The peak calling analysis between the two group (q value ≤ 0.05) were shown in Figure S13 (Supporting Information), including the length of the peak (Figure S13A, Supporting Information), signal value and significance (Figure S13B,C, Supporting Information), enrichment peak annotated to the distribution of functional components from NB and Control group (Figure 8B; Figure S13D, Supporting Information) and the most enriched GO term and pathways in NB versus Control (Figure 8D; Figure S13E, Supporting Information). 0–2 kb around the transcriptional start site (TSS), with such peaks exhibited a high density around the TSS (Figure 8C). Homer known motif search analysis of binding sites revealed that CTCF is significantly enriched (Figure 8E). Notably, IGV visual analysis showed that some regions displayed significantly lower peaks in NB group than in Control, indicating NB reduced transcription of CTCF (Figure 8F). IGV visual analysis of other predicted transcription factors were shown in Figure S14 (Supporting Information). Not surprisingly, CTCF displayed higher content in psoriasis patients versus normal controls detected via immunofluorescence (Figure 8G).

### 2.9 Single‐cell Analysis Reveals the Regulatory Process of CTCF and HSP90AB1 in Psoriasis

**Figure 9:**
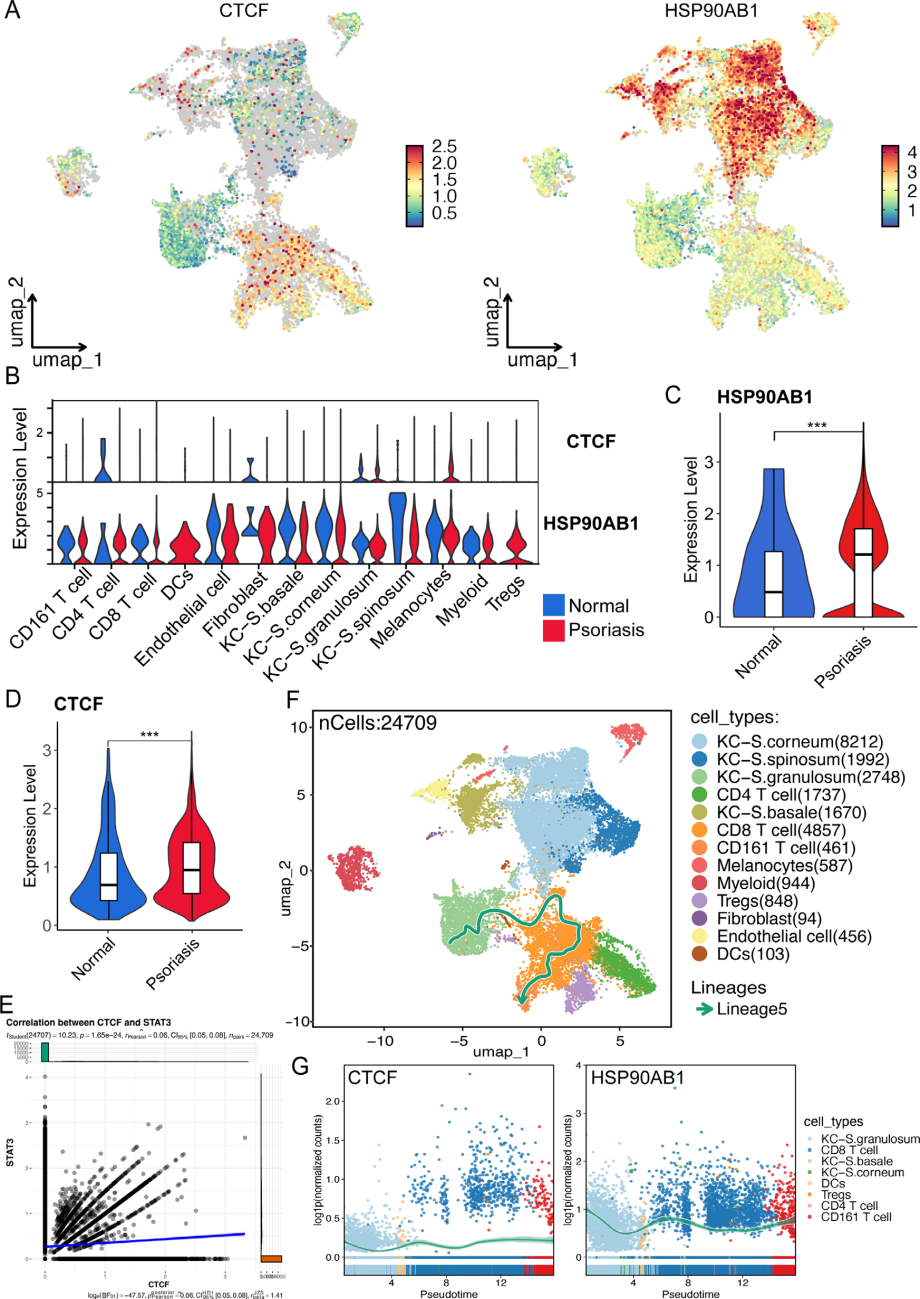
Single‐cell analysis reveals the regulatory process of CTCF and HSP90AB1 in psoriasis. A) Distribution of CTCF and HSP90AB1 in UMAP. B) Expression level of CTCF and HSP90AB1 among different cell populations. C,D) Expression level of CTCF and HSP90AB1 in psoriasis versus normal controls. E) Intercellular correlation assessment of CTCF and STAT3. F) Slinshot pseudotemporal inference of CTCF and HSP90AB. G) Expression patterns of CTCF and HSP90AB1 among KC_S granulosum populations and immune cell populations. Data are presented as mean ± SEM. ***p < 0.001, analyzed by two‐tailed Student's t test.

Here, scRNA‐seq data (GSE151177) was collected, we performed cell screening and data standardization, identified highly variable genes, and normalized for confounding factors. Harmony algorithm was applied, followed by nonlinear dimensionality reduction using UMAP. Distribution of CTCF and HSP90AB1 in UMAP was shown in **Figure** **9**A. Among different cell populations, CTCF and HSP90AB1 both increased in keratinocytes, CD4 T cell and melanocytes (Figure 9B). Consistent with previous results, high expression levels of CTCF and HSP90AB1 were demonstrated in psoriasis (Figure 9C,D). Intercellular correlation assessment also indicated high positive correlation of CTCF and STAT3 indicated (Figure 9E). Slinshot pseudotemporal inference emphasized that expression pattern of CTCF and STAT3 was from keratinocytes to CD161 T cells (Figure 9F). Expression patterns of CTCF and HSP90AB1 among KC_S granulosum populations and immune cell populations were displayed in Figure 9G.

### 2.10 NB Inhibits HSP90AB1 Transcription via CTCF and Leads to Disassemble of the Co‐chaperone Complex

**Figure 10:**
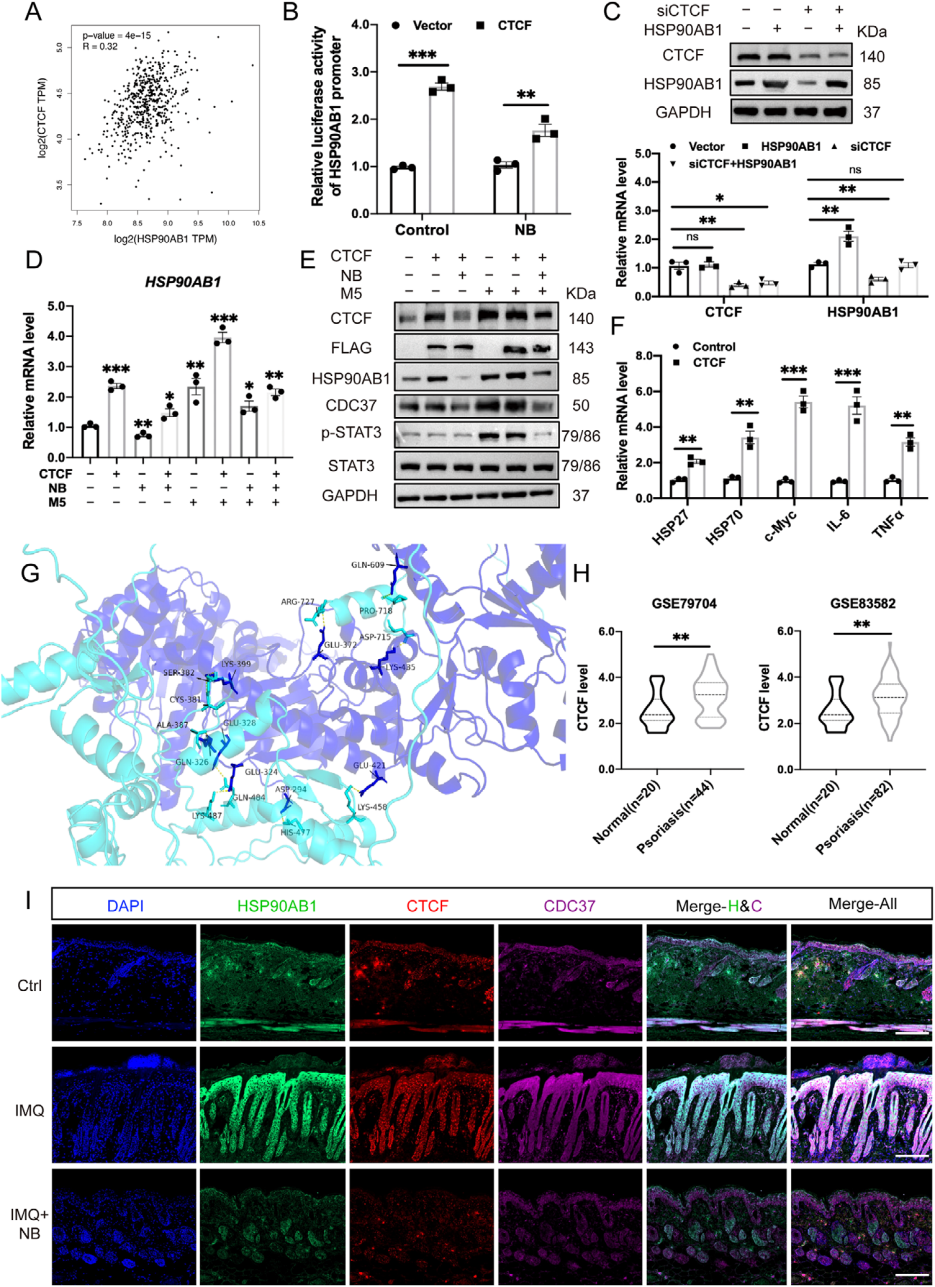
NB inhibits HSP90AB1 transcription via CTCF and leads to disassemble of the co‐chaperone complex. A) Correlation analysis (Pearson) of HSP90AB1 and CTCF basing on GEPIA‐GTEx. B) Luciferase reporter assay of CTCF and *HSP90AB1* promoter. C) Immunoblotting and q‐PCR of CTCF and HSP90AB1 under siCTCF and/or *HSP90AB1* overexpression circumstance. D) The mRNA levels of HSP27, HSP70, c‐Myc, IL‐6 and TNFα in NB versus Control. E) Immunoblotting of CTCF, HSP90AB1, CDC37 and p‐STAT3 under *CTCF* overexpression, M5 or NB treatment. F) The mRNA levels of HSP27, HSP70, c‐Myc, IL‐6 and TNFα after transfection of *CTCF* overexpression plasmid. G) The prediction of the molecular docking site of CTCF (dark blue) and HSP90AB1 (light blue) by Alphafold3. H) CTCF expression levels in GEO databases (GSE79704 and GSE83582). I) Immunofluorescence of HSP90AB1, CTCF, HSP90AB1 of skin sections from Ctrl, IMQ and IMQ+NB groups, scale bar = 100 µm. Data are presented as mean ± SEM (*n* = 3 biologically independent cell samples or 3 mice per group). *p < 0.05, **p < 0.01, ***p < 0.001, ns, no significance, analyzed by two‐tailed Student's t test.

Basing on previous results, we further checked the correlation between HSP90AB1 and CTCF in GEPIA‐GTEx (**Figure** **10**A). To convince that CTCF could trigger *HSP90AB1* transcription, luciferase reporter assay was performed, and we found that overexpression of *CTCF* could increase *HSP90AB1* promoter activity while NB treatment suppresses it to some degree, respectively (Figure 10B). In addition, we co‐transfected HEKa cells with *CTCF* siRNA and *HSP90AB1* overexpression plasmid and the results showed that siCTCF significantly decreased protein level of HSP90AB1 as well as the mRNA level (Figure 10C, quantification results in Figure S12A, Supporting Information). When compared with transfection of *CTCF* overexpression plasmid, M5 treatment also elevated the expression level of CTCF as well as HSP90AB1, CDC37 and p‐STAT3, but could be blocked by NB (Figure 10D,E, quantification results in Figure S12B–E, Supporting Information). The mRNA levels of other relevant genes were also evaluated and not surprisingly, overexpression of *CTCF* induced up‐regulation of HSP27, HSP70, c‐Myc, IL‐6 and TNFα, underlying its possible role in regulating HSPs and responding to inflammation (Figure 10F). Moreover, Alphafold3 was used for the prediction of the molecular docking site of CTCF (dark blue) and HSP90AB1 (light blue) (Figure 10G). We also noticed that CTCF expression levels in GEO databases (GSE79704 and GSE83582) displayed distinct increase in psoriasis patients (Figure 10H). Thus, we detected the immunofluorescence density of CTCF and the colocalization with HSP90AB1‐CDC37 from sections of IMQ mice with or without NB treatment (Figure 10I). As expected, IMQ stimulated CTCF, HSP90AB1 and CDC37 to a large degree, which could be countered by NB. Moreover, the interaction between HSP90AB1 and CDC37 was disrupted under NB but not AUY922 treatment (Figure S12F, Supporting Information). Taken together, what NB does is more than the suppression of HSP90AB1, the co‐chaperone complex which serves as a carrier of multiple clients could also be influenced.

## 3 Discussion

Psoriasis is a complicated and progressive skin disease accompanied by the activation of the immune system response in pathogenesis. Though there are various treatment methods for patients, the limitations of these treatments have already been noted, including adverse effects and treatment resistance.^[^ ^28^ ^]^ TCM like BXP have been found to have promising pharmacological activity against psoriasis since ancient China. Despite their potential in treating psoriasis, the undesirable physicochemical properties (poor permeability, instability, high hydrophilicity or hydrophobicity, and toxicity) and unwanted pharmacokinetics (short half‐life in blood and low bioavailability) limit the clinical studies.^[^ ^24^ ^]^ In our study, we synthesized NB, infinite coordination polymer nanoparticles (BXP‐Fe (III) ICPs) utilizing BXP and Fe^3+^ to enhance the water solubility and bioavailability of BXP and applied NB to ameliorate psoriasis‐like symptoms and inflammation responses either in vivo or in vitro models. Although the number of studies investigating the role of iron in psoriasis is limited, these studies indicate that iron plays a critical role in psoriasis pathogenesis.^[^ ^39^, ^40^ ^]^ Herein, our results demonstrated that NB could significantly increase iron content of the back skin in IMQ mice. By preserving the natural composition of BXP via nanotechnology, we emulate nature's wisdom—harnessing the evolutionarily optimized structural diversity and complexity of natural products. This approach enables multi‐target and synergistic effects that are unattainable with single compounds or simple mixtures.^[^ ^41^ ^]^ Our data support the central tenet of TCM that the bioactivity of BXP is an emergent property of its entire phytochemical matrix, not reducible to the sum of its major constituents. The superior efficacy of the native extract and our iron‐based NB stems from this holistic complexity, where irons act to mimic and stabilize the natural supramolecular assemblies essential for full therapeutic activity.^[^ ^42^ ^]^

To further understand how NB could achieve its therapeutic functions in psoriasis, we performed nascent proteomics and identified HSP90AB1 as a potential key target. HSP90AB1, acting as dimers and binding clients, also known as HSP90beta, is an important member of the large family of HSPs which function as molecular chaperones.^[^ ^35^ ^]^ HSPs are necessary for cellular processes and could promote cell proliferation in cancer, thus, they are targets for new therapeutic approaches in cancer treatment. With the help of co‐chaperones like CDC37, client kinases can be loaded onto HSP90 and form an HSP90‐CDC37‐kinase complex to mediate further activation and maturation. HSP90 client proteins, such as receptor tyrosine kinases JAK1/2 (involved in uncontrolled proliferation), AKT (angiogenesis), and IL‐6 (inflammation), play diverse roles in pathogenesis.^[^ ^43^ ^]^ Therefore, pronounced expression of HSP90 has been detected in almost all classes of cancers as well as benign proliferation like psoriasis.^[^ ^44^ ^]^ Several HSP90 inhibitors have been developed to in treating cancer and plaque‐type psoriasis but none of them have yet been approved due to different drawbacks. It was reported that HSP90 inhibitor RGRN‐305 for oral treatment showed acceptable safety, especially in the low‐dose group, and was associated with clinically meaningful improvement in a subset of patients with plaque psoriasis.^[^ ^21^ ^]^ However, the majority of inhibitors target the N‐terminal domain (NTD) of HSP90, leading to dissociation of heat shock factor‐1 (HSF‐1), which can trigger a compensatory mechanism that induces the transcription of other HSPs, such as HSP70, HSP40, or HSP27, thereby inducing a resistance mechanism called the heat shock response (HSR), which potentially weakens the efficacy of HSP90 inhibitors.^[^ ^45^ ^]^ In our study, we applied AUY922 in IMQ mice as well, which was not superior to NB efficacy. Our results showed that unlike directly inhibiting HSP90 ATPase in the NTD, NB suppressed the expression of HSP90AB1 and blocked the HSP90‐CDC37‐client chaperone cycle, leading to inactivation of STAT3 and Akt which play a pivotal role in psoriasis.^[^ ^46^, ^47^ ^]^ This selective modulation of HSP90 kinase clients, rather than non‐selective inhibition of all the client proteins, could serve as a potential alternative therapeutic strategy for psoriasis. Moreover, we wonder how NB could influence the expression level of HSP90AB1 and hypothesized that the upstream transcription might be the key. Herein, CUT&Tag was performed to investigate the possible transcription factors of HSP90AB1, and we reported that CTCF transcriptionally activating HSP90AB1 and was downregulated by NB. This finding was further corroborated by a luciferase reporter assay, which confirmed that CTCF binds to the *HSP90AB1* promoter. CTCF is known to play a key role in organizing chromatin into highly self‐interacting topologically associated domains (TADs), also acting as a TF controlling the expression of many genes by binding to their TSSs.^[^ ^48^ ^]^ CTCF was involved in a large scale of biological activities, including genome organization, carcinogenesis and inflammation responses.^[^ ^49^, ^50^, ^51^ ^]^ Our study pointed a new mode of function of CTCF in promoting HSP90AB1 transcription in psoriasis. However, the more detailed mechanisms and precise binding sites remain unclear.

In TCM, BXP has been historically prescribed for psoriasis due to its “heat‐clearing” properties, which were also described as removing dampness, detoxifying, and dispelling pathogenic wind. In fact, modern physicians in China believe that the heat and toxicity in blood are the core pathogenesis of psoriasis. In our study, NB could suppress the expression level of HSP90AB1, the heat shock protein family might be mechanistically linked to BXP's “heat‐clearing” therapeutic effects. Unlike reductionist approaches focusing on isolated compounds, our study embraces the holistic nature of herb. By integrating systems biology and nanotechnology, we revealed how BXP collaboratively disrupted the disease networks. To deeply understand the possible connection between the terminology of Chinese and Western medicine, other expression levels of HSPs need to be evaluated in the future research.

Taken together, the present study provides valuable insights into the efficacy of BXP nanoencapsulation to treat psoriasis, representing a promising treatment for psoriasis. Moreover, novel therapeutic target HSP90AB1 and molecular mechanisms for NB in psoriasis treatment were discussed. Nevertheless, the clinical use of BXP is often based on different prescriptions with other TCM according to “*Jun*”‐“*Chen*”‐“*Zuo*”‐“*Shi*” theory,^[^ ^52^ ^]^ leading to the uncertainty of the effective ingredients of TCM, which poses a challenge. While NB shows a promise in IMQ‐induced models, its efficacy in primary keratinocytes or human patients warrants validation. Long‐term safety profiles also require further toxicological assessments as well as the pharmacokinetic and pharmacodynamics studies of NB. To address these questions, future work will focus on the development of mechanisms and theories of TCM, which are crucial steps for the modernization and global acceptance. Nanotechnology, as demonstrated here, is poised to be a pivotal component in this endeavor.

## 4 Conclusion

In conclusion, our findings establish NB as a promising topical nanotherapy for psoriasis, bridging TCM and modern nanotechnology to overcome the pharmacological limitations of BXP. Mechanistically, NB targets the epigenetic regulator CTCF to suppress HSP90AB1 transcription, disrupts the HSP90AB1‐CDC37 chaperone complex, and consequently inactivates the key downstream client proteins STAT3 and Akt, thereby mitigating keratinocyte hyperproliferation and suppressing inflammatory responses. Importantly, NB demonstrates superior efficacy compared to the canonical HSP90 inhibitor AUY922, highlighting its potential as a targeted and safe topical agent. This work unveils the CTCF‐HSP90AB1‐STAT3 axis in psoriasis pathogenesis, offering new insights for future therapeutic development for this chronic dermatological disease.

## Conflict of Interest

The authors declare no conflict of interest.

## Supporting information



Supporting Information

Supporting Information

## Data Availability

The data that support the findings of this study are available in the supplementary material of this article.
