# Supplementary material for: Self‐Assembled Dictamni Cortex Nanoparticles Ameliorate Psoriasis by Epigenetic Modulation of HSP90AB1 and Suppression of the Inflammatory Response
Source: Adv Sci (Weinh). 2025 Oct 20;13(1):e12422. doi: 10.1002/advs.202512422 (PMC12767101; doi:10.1002/advs.202512422)
Supplement: Supplementary file 1 — Supporting Information [file ADVS-13-e12422-s001.docx]

Supporting Information

Self-Assembled *Dictamni Cortex* Nanoparticles Ameliorate Psoriasis by Epigenetic Modulation of HSP90AB1 and Suppression of the Inflammatory Response

Zhengyi Zhang^1#^, Wenqian Du^1#^, Xiaojiang Zhang^2#^, Hongbo Cui^3^, Baochen Cheng^1^, Xiao Han^4^, Ke He^5^, Tingyi Yin^1^, Xinyi Liu^1^, Ningyi Xian^1^, Ziyang Wang^1^, Meng Liu^1^, Dan Han^1^, Jiankang Liu^1,6*^, Yan Zheng^1*^, Ya Wang^2*^

1 Departement of Dermatology, The First Affiliated Hospital of Xi’an Jiaotong University, Xi’an, Shaanxi, 710061, China

2 Center for Gut Microbiome Research, Med-X Institute, The First Affiliated Hospital of Xi’an Jiaotong University, Xi’an, Shaanxi, 710061, China

3 College of Chemistry and Materials Science, Northwest University, Xi’an, Shaanxi, 710127, China

4 Department of Pathogen Biology, School of Basic Medical Science, Xi’an Medical University, Xi’an, Shaanxi, 710021, China

5 Department of Dermatology，Tangdu Hospital, Fourth Military Medical University, Xi’an, Shaanxi, 710032, China

6 School of Health and Life Sciences, University of Health and Rehabilitation Sciences, Qingdao, Shandong, 266071, China

* Correspondence: jkliu@uor.edu.cn (J.L.); zhengyan2018@xjtu.edu.cn (Y.Z.); wangya.0206@foxmail.com (Y.W.)

^#^ These authors contributed equally to this work.

**Materials and Methods**

**Materials and reagents**

Antibodies against NF-κB (#8242), p-NF-κB (#3033), STAT3 (#9139), p-STAT3 (#9145), Akt (#9272), p-Akt (#4060) were purchased from Cell Signaling Technology (Beverly, MA, USA). Antibodies against HSP90AB1 (#T55179), HSP70 (#T55496), CDC37 (#T55967), CTCF(#T56724) were purchased from Abmart (Shanghai, China). Antibodies against GAPDH were purchased from Sigma-Aldrich (#9545, St. Louis, MO, USA). α-Tubulin Recombinant antibody (#80762-1-RR) and Beta Actin Monoclonal antibody (#66009-1) were purchased from Proteintech (Wuhan, China). The dilution factor of the antibodies was ascertained according to the manufacturer’s manual. BXP (YBZ-PFKL-2021003, the National Drug Standards of China and Chinese Drug Administration) was prescribed from The First Affiliated Hospital of Xi’an Jiaotong University. Standards of luteolin (#L107328), quercetin (#Q111273) and wogonin (#W101155) were purchased from Aladdin (Shanghai, China). Standards of obacunone (#T3390), fraxinellone (#T6S0071) and dictamnine (#T5746) were purchased from TargetMol (Shanghai, China). Microfluidic skin organ-on-a-chip was purchased from EACIN BIO​ (Shenzhen, China). Total Iron Colorimetric Assay Kit (#E-BC-K880) was purchased from Elabscience (Wuhan, China). AUY922 (#HY-10215) was purchased from MedChemExpress (Shanghai, China).

**Patient samples**

Psoriasis patients’ datasets related to this article are available in the GEO database (accession #s: GSE13355, GSE114286, GSE53431, GSE79704, GSE83582). Skin tissues (including 8 psoriatic and 8 normal paraffin block) were obtained from the tissue bank of the department of dermatology at the First Affiliated Hospital of Xi’an Jiaotong University with the informed consent waiver and approved by the Ethics Committee of Xi’an Jiaotong University (XJTU1AF2025LSYY-329).

**Preparation of NB**

A 5 mL aliquot of 10 mM Tris-base was placed on a magnetic stirrer. While maintaining rapid agitation (200 rpm), 200 μL of a 10 mg mL⁻¹ BXP suspension was added dropwise. Subsequently, 200 μL of FeCl_3_·6H_2_O solution (20 mg mL⁻¹) and 200 μL of Pluronic F-127 (2%, w/v) were sequentially introduced, each under continuous stirring. The resulting mixture was subjected to ultrasonic treatment (40 kHz, 100 W) for 2 min using a pulse mode (1 s off every 3 s) to ensure homogeneity. Continue stirring for 1-2 days. After that, the MWCO3500 ultrafiltration membrane was used for dialysis or ultrafiltration was carried out with ultrafiltration tubes, so that the remaining system was about 1 mL and stored after ultrasonication. The concentration of BXP in ultrafiltration outer liquid was measured by fluorescence spectrophotometer, and the loading amount was calculated. When used, it is dissolved in 0.9% NaCl aqueous solution and filtered through a 0.45 μm pore size filter membrane and can be directly injected locally after filtration.

**Characterization of NB**

The sizes and morphologies of the samples were determined by transmission electron microscope (Thermo Fisher Scientific, Talos-L120C G2, USA). The composition was obtained by Lorenz transmission electron microscope (Thermo Fisher Scientific, Talos F200X, USA) with an energy-dispersive spectroscopy (EDS) system. The loading amount was calculated via Fluorescence spectrophotometer (Hitachi, F-7100, Japan). The average size and zeta potential were determined through dynamic light scattering (Malvern Zetasizer Nano-ZSP system, ZEN5600, U.K.). X-ray photoelectron spectroscopy (XPS) spectra were obtained by X-ray photoelectron spectrometer (Thermo Fisher Scientific, ESCALAB Xi+, USA). Ultraviolet-visible (UV–vis) absorption spectra were obtained using ultraviolet spectrophotometer (Hitachi, U-3900, Japan). Fourier transform infrared spectroscopy (FTIR) analysis was performed on a micro-infrared spectrometer (Bruker, VERTEX70, German). Inductively coupled plasma mass spectrometry (ICP-MS) data were recorded from inductively coupled plasma mass spectrometer (P.E., NexION 350D, USA).

***In vitro* drug release**

The dialysis bag diffusion method was used to assess the in vitro drug release behavior of NB. The NB were dispersed in 5 mL of deionized water and transferred to a dialysis bag. After sealing, the mixture was put into a PBS solution of pH 7.4 containing 5% polysorbate 80 and shaken in a 37 °C H_2_O bath at 100 g. Then, 1 mL samples were taken out from the receptor compartment at different time points and added to an equal volume of fresh release medium. The absorbances of the collected samples were determined in a Fluorescence spectrophotometer, and the cumulative percentage release of BXP from NB was calculated.

**Mice**

All mouse experiments were performed according to the protocol approved by the laboratory animal welfare ethics committee of Xi’an Jiaotong University (XJTUAE2023-2292). C57BL/6J male mice (8 – 10 weeks of age) were randomly divided into four groups (*n* = 6 mice per group), namely, a vehicle-treated control, an IMQ group, an IMQ + NB group, and an IMQ + AUY922 group. NB (20 mg kg^-1^)^[1]^ and AUY922 (50 mg kg^-1^)^[2]^ were subcutaneously injected 30 min before IMQ application. A daily topical dose of 62.5 mg of IMQ cream (5%, Mingxin, Chengdu, China) was applied to the shaved backs of the mice for 5 consecutive days, and the mice were sacrificed on day 6. A scoring system was used to score skin inflammation based on PASI.^[3]^

**Cell lines**

HEKa cells (#PCS-200-011, Primary Epidermal Keratinocytes; Normal, Human, Adult), with great biological relevance to this study, were obtained from ATCC (Manassas, VA, USA) and cultured in high-glucose DMEM supplemented with 10% fetal bovine serum (Gibco, #10270‐106) and 1% penicillin–streptomycin (Beyotime, #ST488).^[4]^ HEKa cells were not included in Cellosaurus since its genetic and phenotypic background may change, they could retain physiological responsiveness and stability for psoriatic modeling and mimic *in vivo* situation better than HaCaT cells, ensuring consistency of conclusions. The cells (passage = 4) were contamination free and cultured at 37 ℃ in a humidified-atmosphere incubator containing 5% CO_2_ for further treatments. *In vitro* psoriatic model induced by M5 (a cocktail of cytokines, including TNF-α, IL-17A, IL-22, IL-1α, and Oncostatin-M, 10 ng mL^-1^) (Peprotech, Cranbury, NJ, USA) was described as before.^[5]^ All reagents and procedures were strictly sterile, and all operations were completed in a biological safety cabinet.

**Molecular docking**

The molecular structures of the core ingredients (wogonin, luteolin, quercetin, fraxinellone, obacunone, dictamnine) were obtained from PubChem (<https://pubchem.ncbi.nlm.nih.gov/>), and energy minimization was performed using Chem3D software. 3D X-ray crystal structures of the key targets (HSP90AB1, TNFα, IL-6) were obtain from the PDB database (http:// [www.rcsb.org](http://www.rcsb.org)). Both ingredient and protein files were converted into PDBQT format. Molecular docking was performed using AutodockVina *v1.2.2* software and the binding energy results were obtained. Finally, the docking results were visualized using PYMOL software.

**Metabolic labeling of nascent proteome**

About 2 × 10^7^ HEKa cells were pre-incubated with the serum-free DMEM lacking methionine (Sigma-Aldrich, #D0422) for 2 h, and then incubated with DMEM containing 0.4 mM azidohomoalanine (Aha) for 4 h. Cells were harvested using protein extraction buffer (8 M urea, 100 mM NH_4_HCO_3_, 0.1% Triton X-100 and complete protease inhibitor cocktail 1 ×, pH 7.5). The click reaction was performed overnight with 2 mM alkynylated biotin in click reaction buffer (TBTA: 100 μM, CuSO_4_: 4 mM, TCEP: 2 mM) and after the click reaction, five volumes of precipitation buffer (acetone: alcohol: acetic acid = 50:50:1) were add to the samples and incubated at −20 °C overnight. Non-precipitate was removed by centrifugation at 20,000 × g, 4 °C for 30 min. The precipitate was subsequently washed with ice-cold acetone and 75% ice-cold ethanol and resuspended in 8 M urea in 100 mM NH_4_HCO_3_. Nascent proteome was enriched by streptavidin magnetic beads (Pierce, #88816) on a rotator at 4 °C overnight.

**LC-MS/MS and standard quantification**

BXP were extracted with methanol in an ultrasonic bath for 30 min. The extract was filtered, and the residue was further extracted twice as before, all three filtrates were combined. The sample was store at 4 ℃ for subsequent study. The analysis was performed on a Thermo Scientific^TM^ Q Exactive^TM^ Plus Hybrid Quadrupole-Orbitrap^TM^ Mass Spectrometer coupled to a Vanquish^TM^ Horizon UHPLC system (Thermo Fisher Scientific, USA). Chromatographic separation was achieved on an ACQUITY UPLC BEH C18 column (100 mm × 2.1 mm, 1.7 µm, Waters, UK) maintained at 35 °C. The mobile phase consisted of (A) water containing 0.1% (v/v) formic acid and (B) acetonitrile. The gradient elution program was set as follows: 0 – 2 min, 5% B; 2 – 6 min, 5% to 20% B; 6  – 12 min, 20% to 50% B; 12 –16 min, 50% to 80% B; 16 – 17 min, 80% to 95% B; 17 – 20 min, 95% B; followed by re-equilibration at 5% B for 4.9 min. The flow rate was 0.2 mL/min, and the injection volume was 2 µL. High-purity reference standards of six major bioactive compounds in BXP (luteolin, quercetin, wogonin, dictamnine, fraxinellone, and obacunone) were accurately weighed and dissolved in methanol to prepare individual stock solutions (1 mg mL^-1^). A series of working standard solutions at different concentrations were prepared by appropriate dilution of the stock solutions. The quantitative analysis was performed using the external standard method. Standard curves for each compound were constructed by plotting the peak area against the corresponding concentration of the analytical standards. All calibration curves exhibited excellent linearity (R^2^ > 0.99) over the tested concentration ranges. The content of each compound in the BXP extract was then be calculated by interpolating the measured peak area of the sample into its respective standard curve.

**CUT&Tag**

HEKa cells are first mixed with magnetic beads, and then the target protein-specific antibody (HSP90AB1 antibody) is incubated. To amplify the signal, secondary antibody incubation was followed. Finally, the pAG-Tn5 transposome is incubated to allow the transposome to enter the cell and bind to the antibody. Then Mg^2+^ is added to activate the cleavage activity of the Tn5 enzyme and interrupt the binding. The DNA is extracted and amplified to construct the library. The PCR products were purified by 1.3X magnetic beads, and the library was tested for fragment range and effective concentration by Quant-iTTM dsDNA HS analysis kit (Invitrogen, MA, USA) and qPCR on an Agilent 2100 analyzer (Agilent Technologies). Qualified libraries were sequenced on Illumina or GeneMind, and the sequencing strategy was paired-end 150 bp sequencing (PE150).

**Dimension reduction and clustering analysis**

First, the Seurat R package was employed to convert scRNA-seq data into Seurat format.^[6]^ Consistent with public data quality control, we performed cell screening and data standardization, identified highly variable genes, and normalized for confounding factors. The optimal principal components were then selected using the ‘ElbowPlot’ and ‘JackStraw’ methods. For multi-sample analysis, the Harmony algorithm was applied, followed by nonlinear dimensionality reduction using UMAP. Cell populations were identified through neighborhood network and cluster analysis with a resolution of 0.1. The visualization of cell clustering distribution was achieved using the “Seurat”, “ggplot2” and “SCP” R packages. Differentially expressed genes were identified using the ‘FindAllMarkers’ and cell clusters were annotated based on the expression of typical marker genes. In the analysis and visualization of intercellular correlations, the R-package ggcorrplot2 was used for correlation assessment, and the Pearson rank correlation coefficient was adopted as a statistical method to analyze the degree of intercellular association.

**Cell trajectory inference**

Cell trajectory inference was also performed using the SCP package, which is specifically designed for analyzing scRNA-seq data. The primary aim of Slingshot is to reveal dynamic cellular changes by inferring the developmental trajectory or pseudo-temporal sequence of cells.^[7]^

**The luciferase reporter assay**

Briefly, the HEKa cells were plated in 12-well plates at a density of 1 × 10^5^ cells per well then transfected with 0.5 μg of a promoter-luciferase plasmid. To normalize the transfection efficiency, the cells were also co-transfected with 5 ng of pRL-CMV (Renilla luciferase). PEI was used for the transfection. After 48 h, the luciferase activity was measured via Dual-Luciferase Assay kit (Promega). The results were obtained from three independent experiments. The primer applied for cloning HSP90AB1 promoter was described in **Supplementary Table S4**.

**RNA Extraction and Quantitative PCR Analysis**

Total RNA from skin tissues and HEKa cells was extracted by TRIzol reagent (InvitrogenTM, Carlsbad, CA, USA). cDNA was synthesized through an RT- PCR kit (TaKaRa, Dalian, China) and subjected to a quantitative real-time PCR with primers (listed in **Supplementary Tables S3**). Gene expression value was normalized to *GAPDH/Gapdh* and analyzed using the 2^−∆∆Ct^ method.

**Protein Extraction and Immunoblotting**

Skin tissues and cell lysates were prepared in ice-cold IP lysis buffer (Beyotime, Shanghai, China) containing protease inhibitor cocktail (MCE, #HY‐K0011). Equal aliquots (10 μg for cells or 20 μg for tissues) of the protein samples were separated by 8–12% SDS-PAGE and transferred onto polyvinylidene difluoride (PVDF) membranes (Millipore, MA, USA, #IPVH00010). Membranes were blocked with 5% nonfat milk in 1 × TBST buffer for 1 h at room temperature and then incubated with primary antibodies at 4 ℃ overnight. After three 1 × TBST washing procedures (lasting 10 min, 3 times), the membranes were incubated with horseradish-peroxidase-conjugated secondary antibodies (1:3000) for 1 h at room temperature. Chemiluminescence was achieved using an ECL Western blotting detection kit (Pierce, Rock- ford, IL, USA), and the results were quantified using ImageJ (National Institutes of Health, Bethesda, MD, USA) to obtain the optical density ratio of target proteins relative to GAPDH.

**Plasmids and siRNA**

*HSP90AB1* (#P60128) and *CTCF* (#P57047) overexpression plasmids were purchased from Miaoling Biotechnology (Hubei, China). siRNA targeting *CTCF* and scrambled siRNA was used as negative control. The transfection was performed using TransIT-X2® Dynamic Delivery System (Amresco, USA), as described in the supplier’s manual. Detailed siRNA targeting sequences are listed in **Supplementary Table S3**. HEKa cells were prepared in six-well plates for transfection. siRNA (100 nmol), plasmids (2 μg) and TransIT-X2 (1:3) were incubated in OPTI-MEM for 15 min and the mixture was then added to HEKa cells for 10 h. The medium was replaced with H-DMEM for another 48 h, and then the cells were treated with different conditions.

**H & E and Immunohistochemical Staining**

H & E staining and immunohistochemistry were performed according to the standard protocol as previously described.^[8]^ Briefly, after the sacrifice, skin samples were isolated and fixed in 4% formaldehyde. Then, the samples were embedded in paraffin and cut into 5 μm thick sections. The dilution factor of the antibody was determined according to the manufacturer’s instructions.

**Immunofluorescent Staining**

Skin tissues were fixed in 4% PFA for 24 h and dehydrated in 30% sucrose solution for another 24 h. The dehydrated tissues were embedded in OCT (SAKURA, 4583) and frozen at − 80 °C for 2 h. Frozen tissues were sectioned at a thickness of 7 µm. Then the sections were placed in the citrate antigen retrieval solution for heat‐induced antigen retrieval. Sections were blocked in 10% donkey serum for 1 h at room temperature, then incubated with primary antibodies overnight at 4 °C according to suggested dilution factor, after wash, secondary antibody incubation lasted for 1 h at room temperature. Sections were mounted using mounting medium containing DAPI (Vector, H‐1200). Image acquisition was performed at on an Olympus microscope.

**Statistical Analysis**

Statistical analyses were performed in Prism 8 for macOS, version 8.2.1 (GraphPad Software, CA, USA). Data were represented as the mean ± SEM. Sample size (*n*) for each statistical analysis was included in each figure illustration. When two groups were compared, the statistical significance was analyzed using a two-tailed unpaired Student’s t-test. One-way ANOVA followed by Bonferroni’s post hoc analysis was used to assess differences between more than two groups. Results were considered statistically significant at *p* value less than 0.05. **p* < 0.05, ***p* < 0.01, ****p* < 0.001, ns, no significance.

***Supplementary Table S2 Standard Quantification of*** ***Six*** ***Active Components from BXP***

**Standard Quantification of Six Active Components from BXP (330 mg L^-1^)**

| **Name** | **Molecular Formula** | **Calc. MW** | | **Content (μg L^-1^)** | |
| --- | --- | --- | --- | --- | --- |
| luteolin | C15 H10 O6 | 287.05501 | 156.90035 | |  |
| obacunone | C26 H30 O7 | 455.2064 | 38.204168 | |  |
| quercetin | C15 H10 O7 | 303.0499 | 48.19768 | |  |
| wogonin | C16 H12 O5 | 285.0758 | 19.188488 | |  |
| dictaminine | C12 H9 N O2 | 200.0706 | 25.922205 | |  |
| fraxinellone | C14 H16 O3 | 233.1172 | 14.803918 | |  |

***Supplementary Table S3*** ***Primers of qRT-PCR***

**Appendix 1. Primer Sequences (*homo species*)**

| **Primer** | | **Sequence (5’→3’)** |
| --- | --- | --- |
| HSP90AB1 | Forward | CATCTCCATGATTGGGCAGTT |
|  | Reverse | CTTTGACCCGCCTCTCTTCTA |
| HSP70 | Forward | AGTGATGGATGCAACACAGATT |
|  | Reverse | CCAATGTCGTGTCAAATGCAG |
| IL-17A | Forward | TCCCACGAAATCCAGGATGC |
|  | Reverse | GGATGTTCAGGTTGACCATCAC |
| IL-17F | Forward | GCGTTTCCATGTCACGTAACA |
|  | Reverse | CAGCCCAAGTTCCTACACTGG |
| IL-1α | Forward | TGGCTCATTTTCCCTCAAAAGTTG |
|  | Reverse | AGAAATCGTGAAATCCGAAGTCAAG |
| IL-1β | Forward | CCAGGGACAGGATATGG AGCA |
|  | Reverse | CCAGGGACAGGATATGG AGCA |
| TNF-α | Forward | TCCTTCAGACACCCTCAACC |
|  | Reverse | TCCTTCAGACACCCTCAACC |
| HO-1 | Forward | AAGACTGCGTTCCTGCTCAAC |
|  | Reverse | AAAGCCCTACAGCAACTGTCG |
| NQO1 | Forward | GAAGAGCACTGATCGTACTGGC |
|  | Reverse | GGATACTGAAAGTTCGCAGGG |
| SOD1 | Forward | GGTGGGCCAAAGGATGAAGAG |
|  | Reverse | CCACAAGCCAAACGACTTCC |
| SOD2 | Forward | GGAAGCCATCAAACGTGACTT |
|  | Reverse | CCCGTTCCTTATTGAAACCAAGC |
| c-Myc | Forward | GTCAAGAGGCGAACACACAAC |
|  | Reverse | TTGGACGGACAGGATGTATGC |
| CTCF | Forward | CAGTGGAGAATTGGTTCGGCA |
|  | Reverse | CTGGCGTAATCGCACATGGA |
| HSP27 | Forward | ACGGTCAAGACCAAGGATGG |
|  | Reverse | AGCGTGTATTTCCGCGTGA |
| GAPDH | Forward | ACAACTTTGGTATCGTGGAAGG |
|  | Reverse | GCCATCACGCCACAGTTTC |

**Appendix 2. Primer Sequences (*mus musculus*)**

| **Primer** | | **Sequence (5’→3’)** |
| --- | --- | --- |
| Hsp90ab1 | Forward | GTCCGCCGTGTGTTCATCAT |
|  | Reverse | GCACTTCTTGACGATGTTCTTGC |
| Il-1α | Forward | CGAAGACTACGTTCTGCCATT |
|  | Reverse | GACGTTTCAGAGGTTCTCAGAG |
| Il-1β | Forward | GCTTCAGGCAGGCAGTATCA |
|  | Reverse | CTCTGCTTGTGAGGTGCTGA |
| Il-6 | Forward | CACTTCACAAGTCGGAGGCT |
|  | Reverse | GCCACTCCTTCTGTGACTCC |
| Tnf-α | Forward | ATGAGCACAGAAAGCATGATC |
|  | Reverse | TACAGGCTTGTCACTCGAATT |
| Il-17a | Forward | TACCTCAACCGTTCCACGTC |
|  | Reverse | TTTCCCTCCGCATTGACACA |
| Il-17f | Forward | TGCTACTGTTGATGTTGGGAC |
|  | Reverse | AATGCCCTGGTTTTGGTTGAA |
| Il-22 | Forward | GGTGTCTTGTGGCCTCCTATG |
|  | Reverse | ATAGCCATCGGGACACCAGG |
| Il-23 | Forward | ATGCTGGATTGCAGAGCAGTA |
|  | Reverse | ACGGGGCACATTATTTTTAGTCT |
| Gapdh | Forward | CTGACTTCAACAGCGACACC |
|  | Reverse | TAGCCAAATTCGTTGTCATACC |

***Supplementary Table S4 Primers of Luciferase and siRNA***

| **Primer** | | **Sequence (5’→3’)** |
| --- | --- | --- |
| HSP90AB1 promoter | Forward | CGCGGGTACCGACACAGAAAGGATCCAGCTC |
|  | Reverse | CGCAAAGCTTTAGATCCCTCCGCCTTAAAGG |
| CTCF-siRNA#1 | - | GCAAGAAUGUCCAGAACAATT |
| CTCF-siRNA#2 | - | ACAAACUGCACUGAAACGGAC |
| Control siRNA | - | UUCUCCGAACGUGUCACGUTT |

***Supplementary Figure S1 Classification of Major Compounds of BXP***

***
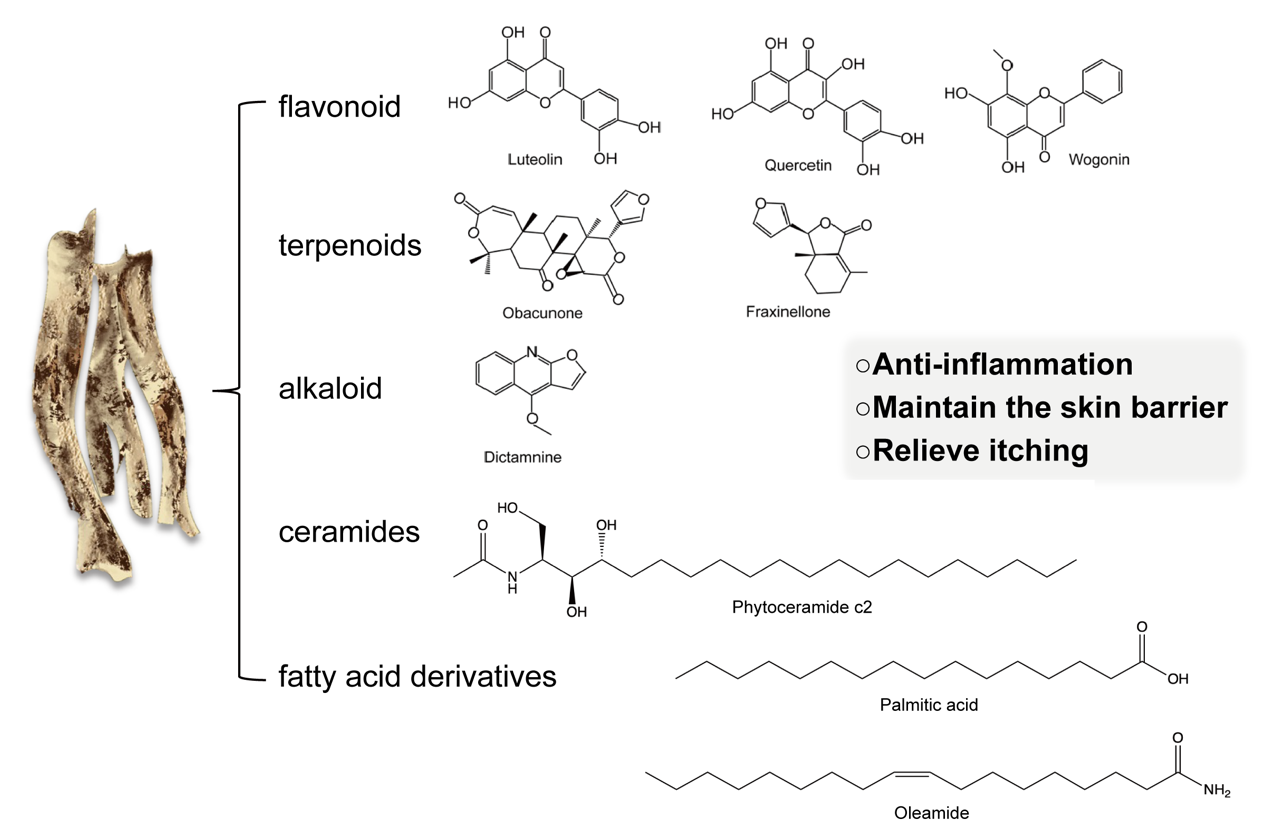
***

***Supplementary*** ***Figure S2*** ***Q Exactive^TM^ Plus Detection of BXP and Six Components***

**
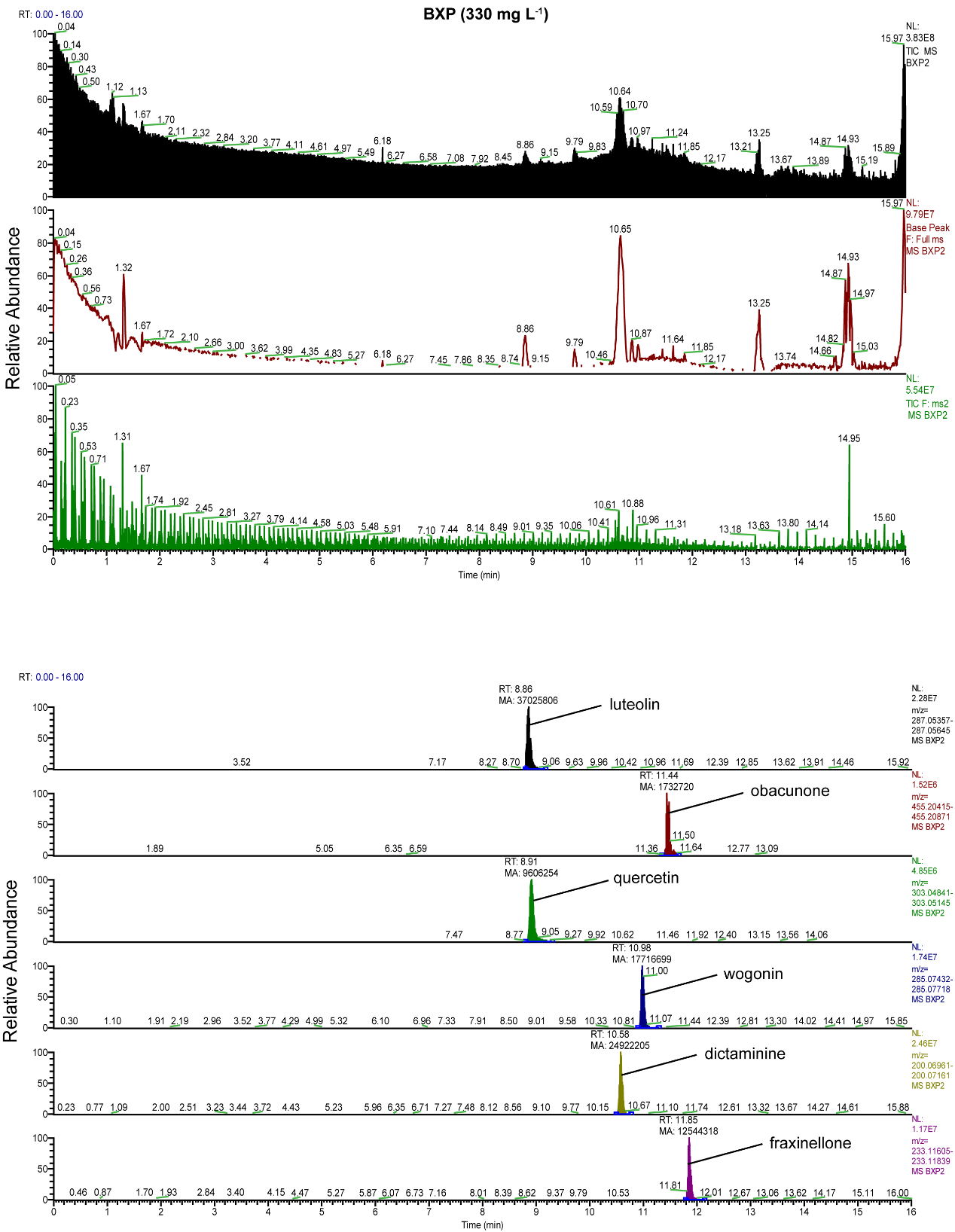
**

**Figure S2. Q Exactive^TM^ Plus detection of BXP and six components.** The whole spectrum of BXP (up). The chromatograms of six components (down).

***Supplementary Figure S3 The Spectrums and Standard Curves of Six Components***

***
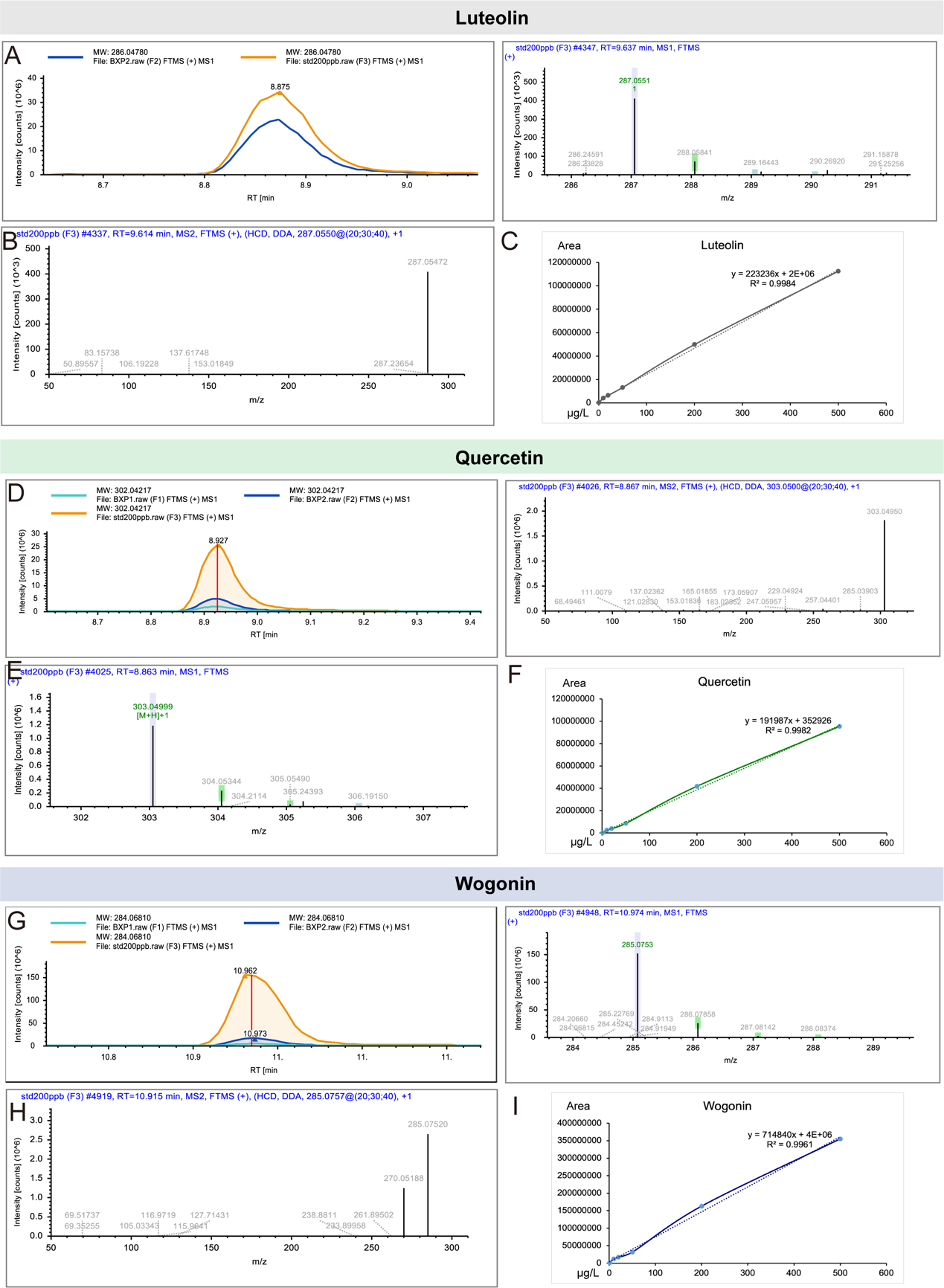
***

**Figure S3. The spectrums and** **standard curves of six components. A)** MS1 spectrum, (**B**) MS2 spectrum and standard curve (**C**) of luteolin. **D)** MS1 spectrum, (**E**) MS2 spectrum and standard curve (**F**) of quercetin. **G)** MS1 spectrum, (**H**) MS2 spectrum and standard curve (**I**) of wogonin.

***
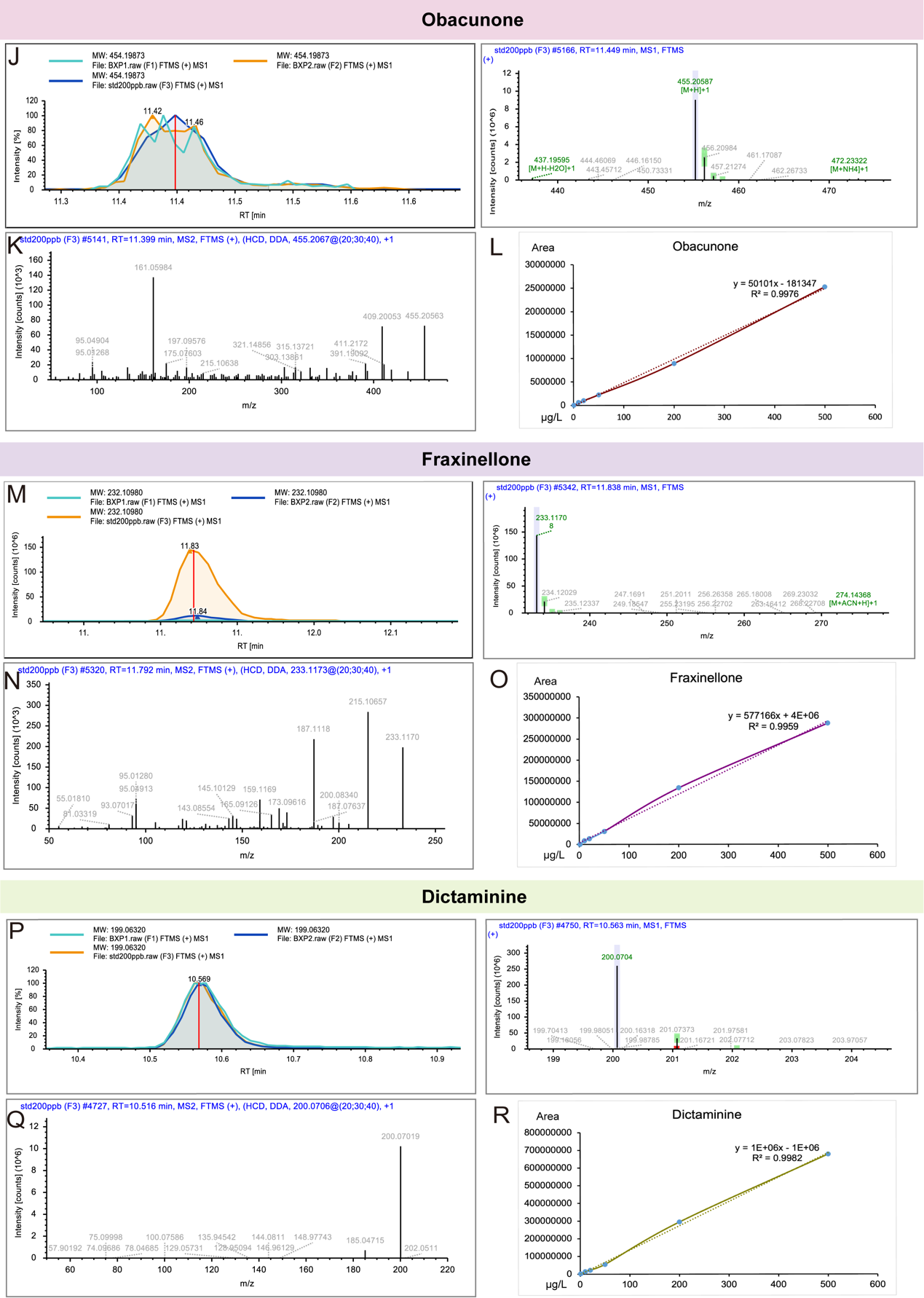
***

**Figure S3. The spectrums and standard curves of six components. J)** MS1 spectrum, (**K**) MS2 spectrum and standard curve (**L**) of obacunone. **M)** MS1 spectrum, (**N**) MS2 spectrum and standard curve (**O**) of fraxinellone. **P)** MS1 spectrum, (**Q**) MS2 spectrum and standard curve (**R**) of dictamnine.

***Supplementary Figure S4*** ***The MS1 Spectrums of Representative Fatty Acid Derivatives and Ceramides***

***
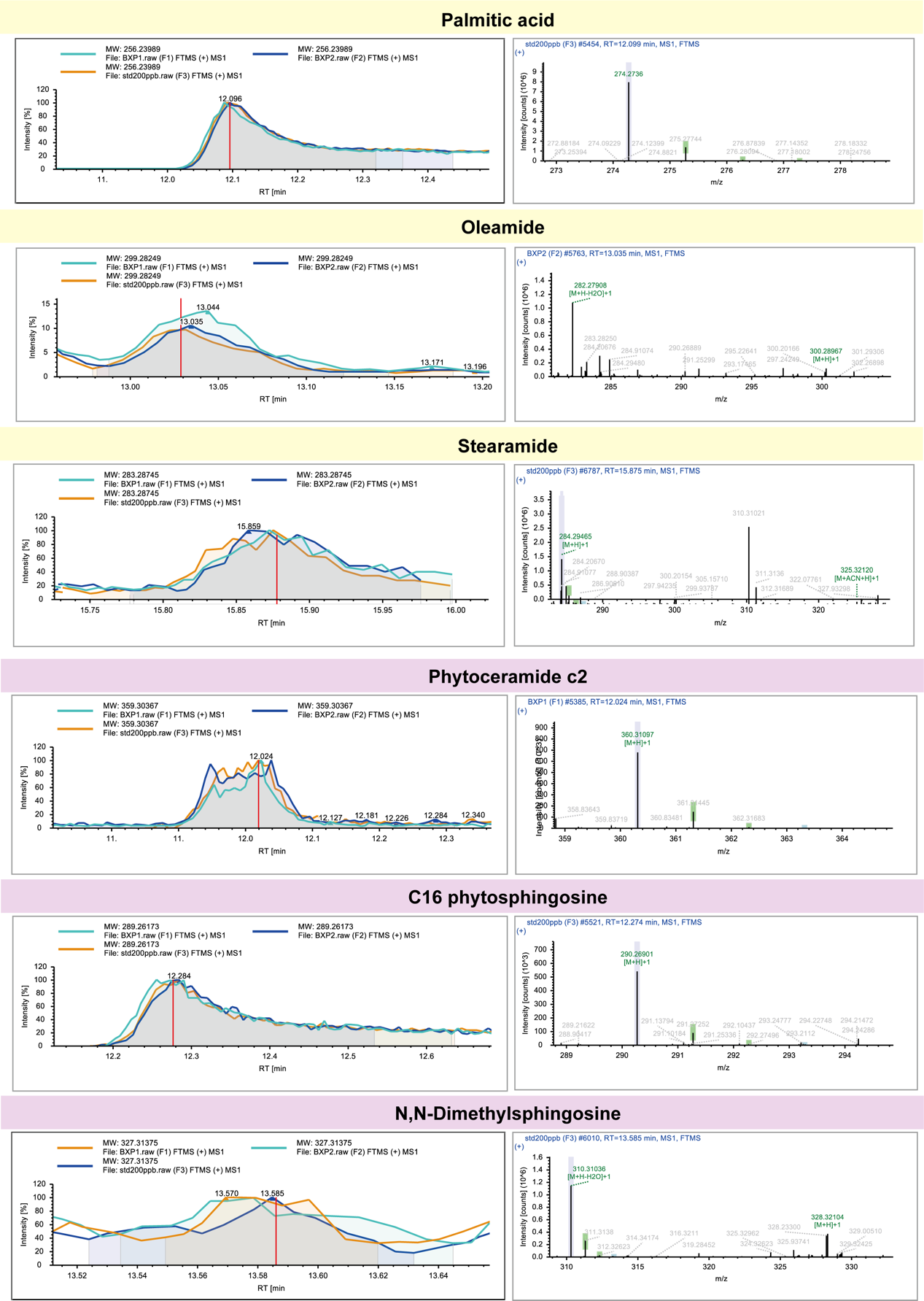
***

**Figure S4. The MS1 spectrums of representative fatty acid derivatives and ceramides.**

***Supplementary Figure S5 Cell Viability and Quantitative Results***

*
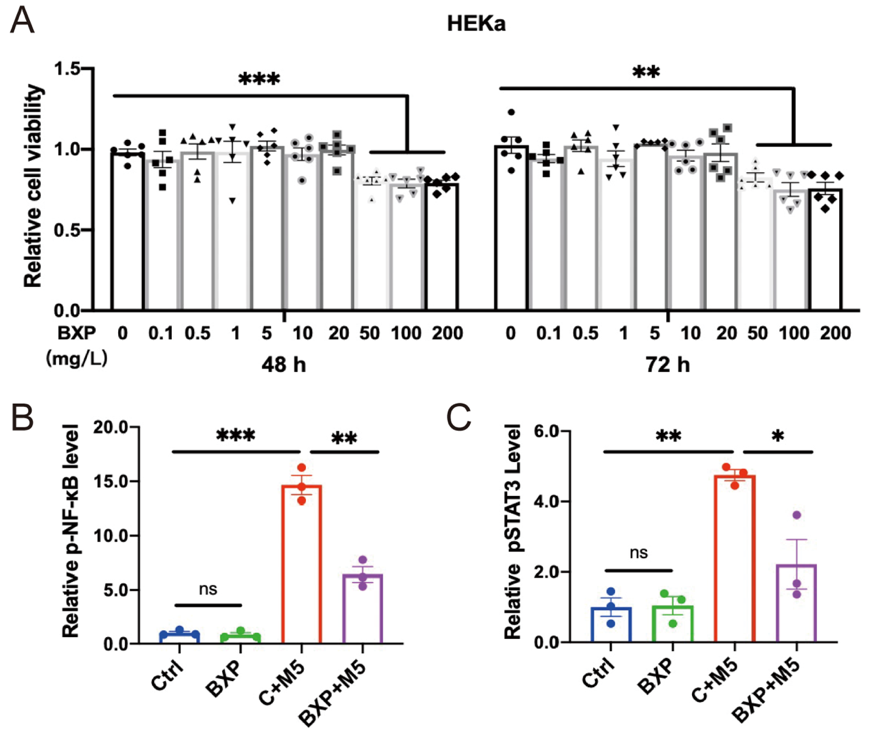
*

**Figure S5. Cell viability and quantitative results. A)** Cell viability of HEKa cells under treatment of different concentrations of BXP after 48 h and 72 h. **B-C)** Quantification results of the immunoblotting. (Two-tailed Student’ s t test was used for the statistical analysis. Data are presented as means ± SEM (*n* = 3 or 6 biologically independent cell samples). **p* < 0.05, ***p* < 0.01, ****p* < 0.001, ns, no significance)

***Supplementary Figure S6***

***
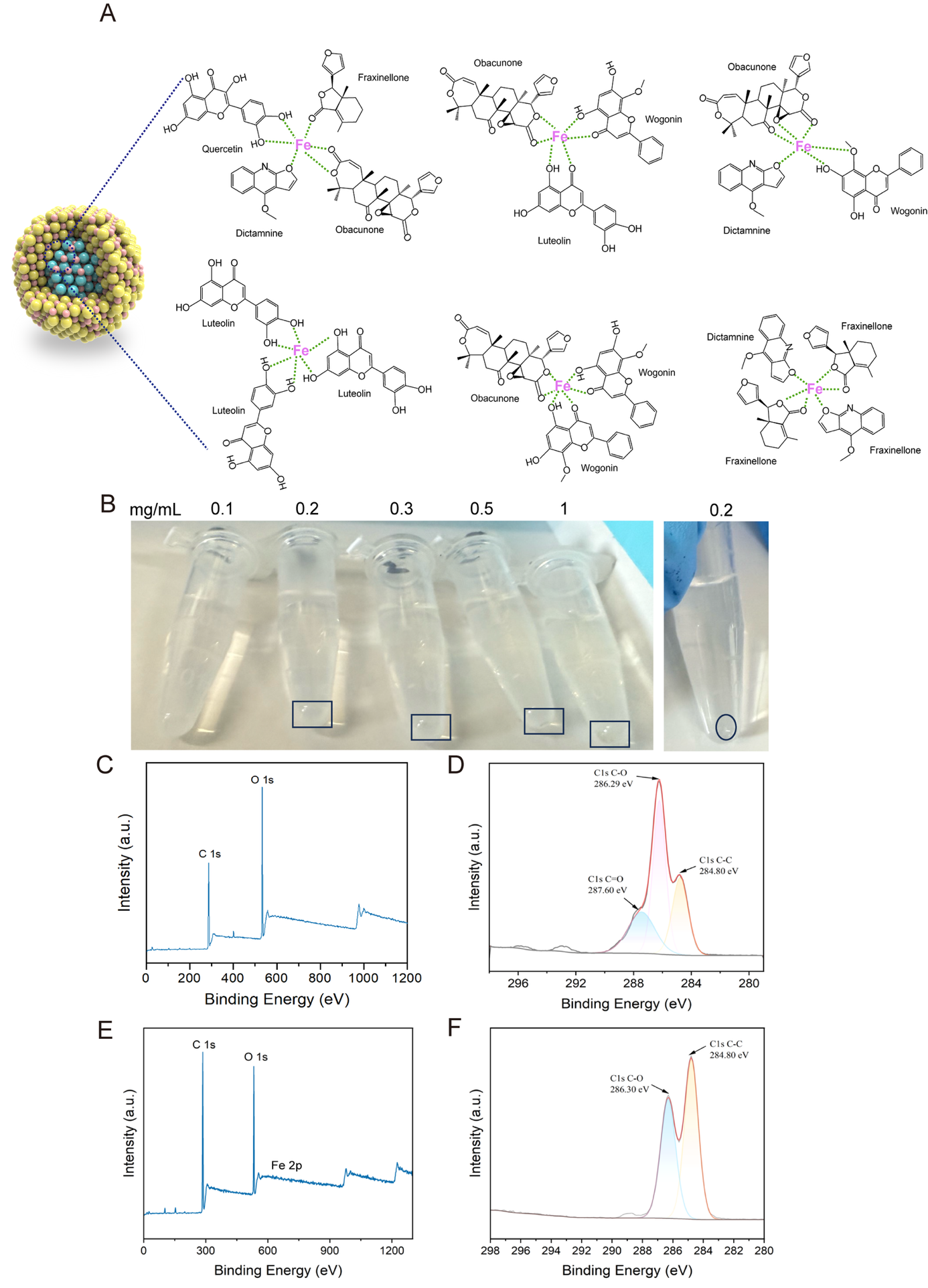
***

**Figure S6.** **A)** The self - assembling of BXP and Fe^3+^ (Only the potentially existing coordination structures are presented). **B)** The aqueous solubility of BXP under different concentrations (0.1, 0.2, 0.3, 0.5, 1 mg mL^-1^). The XPS survey spectrum of BXP (**C**) and NB (**D**) and high resolution XPS spectra of C 1s of BXP (**E**) and NB (**F**).

***Supplementary Figure S7 CCK-8 and Immunoblotting Analysis of*** ***Different Combinations of Six Components***

***
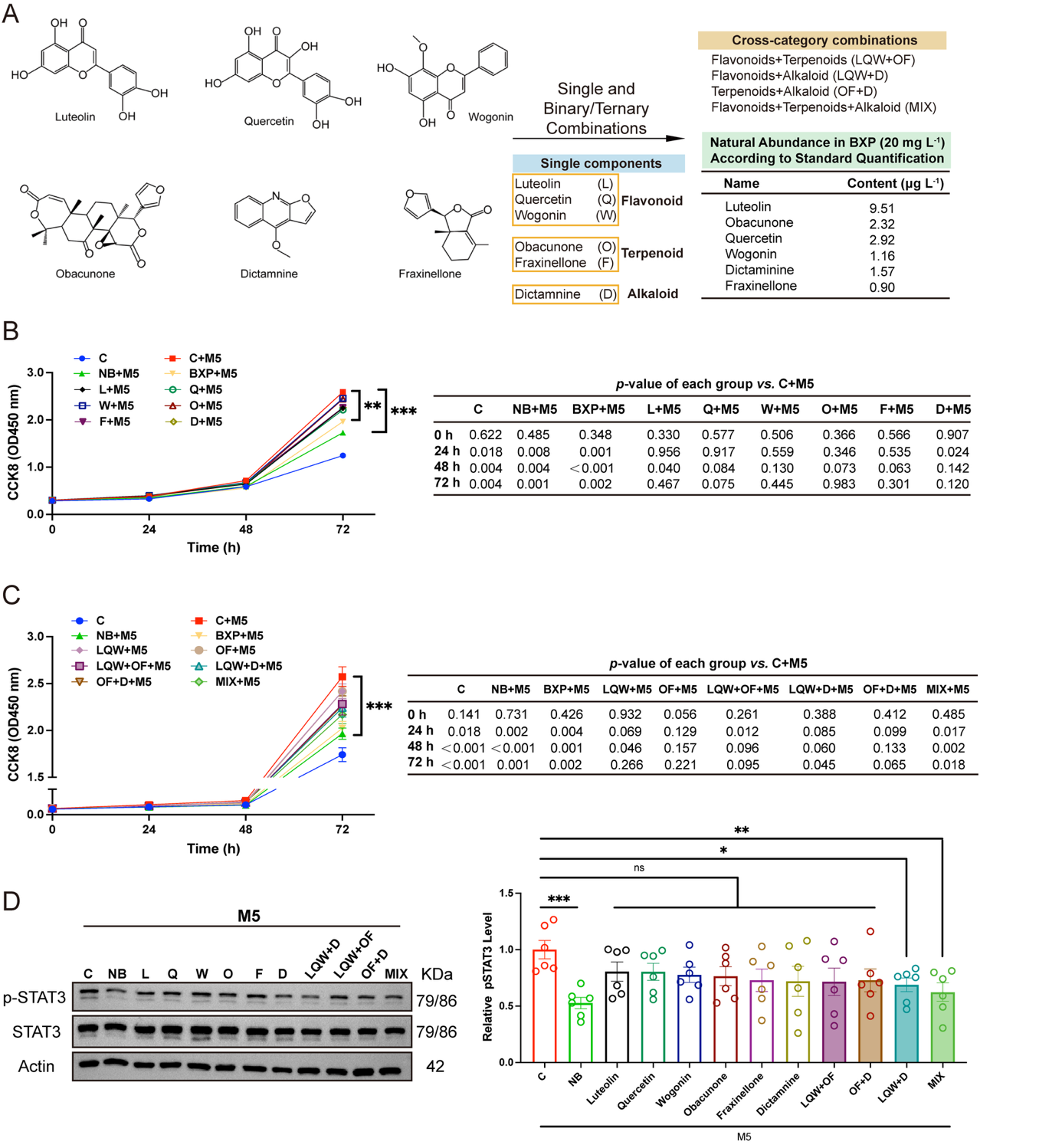
***

**Figure S7. CCK-8 and immunoblotting analysis of different combinations of six components. A)** Grouping through single /cross-category combinations and calculated natural abundance in BXP (20 mg L^-1^) according to standard quantification (**Supplementary Table S2**). Cell proliferation under M5 treatment and different intervention groups (single component (**B**)) and (combined components (**C**)) via CCK-8 assay, and *p*-value of each group *vs.* C+M5 within 72 h. **D)** Immunoblotting of p-STAT3 of groups from single and combined components, and quantification results of the immunoblotting. Data are presented as means ± SEM (*n* = 6 biologically independent cell samples). **p* < 0.05, ***p* < 0.01, ****p* < 0.001, ns, no significance, analyzed by two-tailed Student’s t test.

***Supplementary Figure S8 Quantification Results of The Immunoblotting***

**
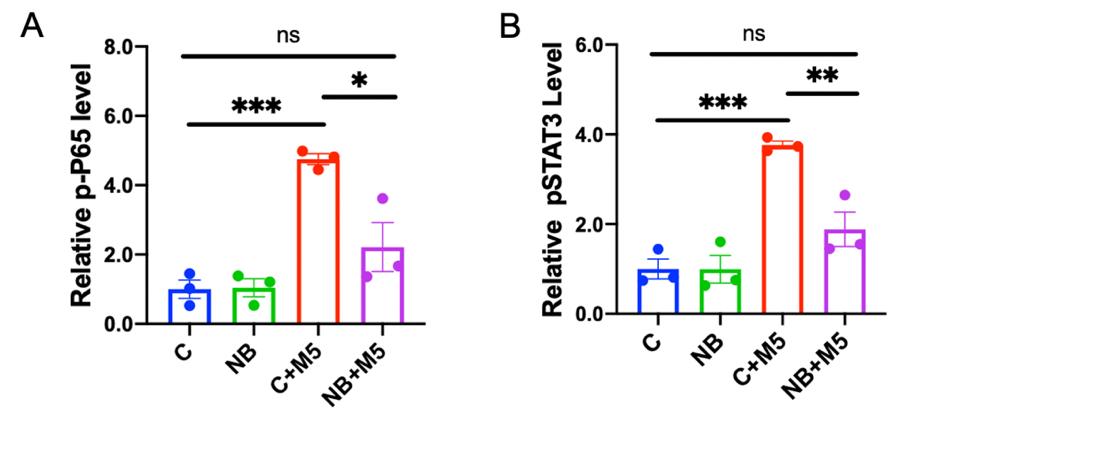
**

**Figure S8. Quantification results of the immunoblotting. A)** Relative level of p-P65. **B)** Relative level of p-STAT3. (Two-tailed Student’ s t test was used for the statistical analysis. Data are presented as means ± SEM (*n* = 3 biologically independent cell samples). **p* < 0.05, ***p* < 0.01, ****p* < 0.001, ns, no significance)

***Supplementary Figure S9***

***
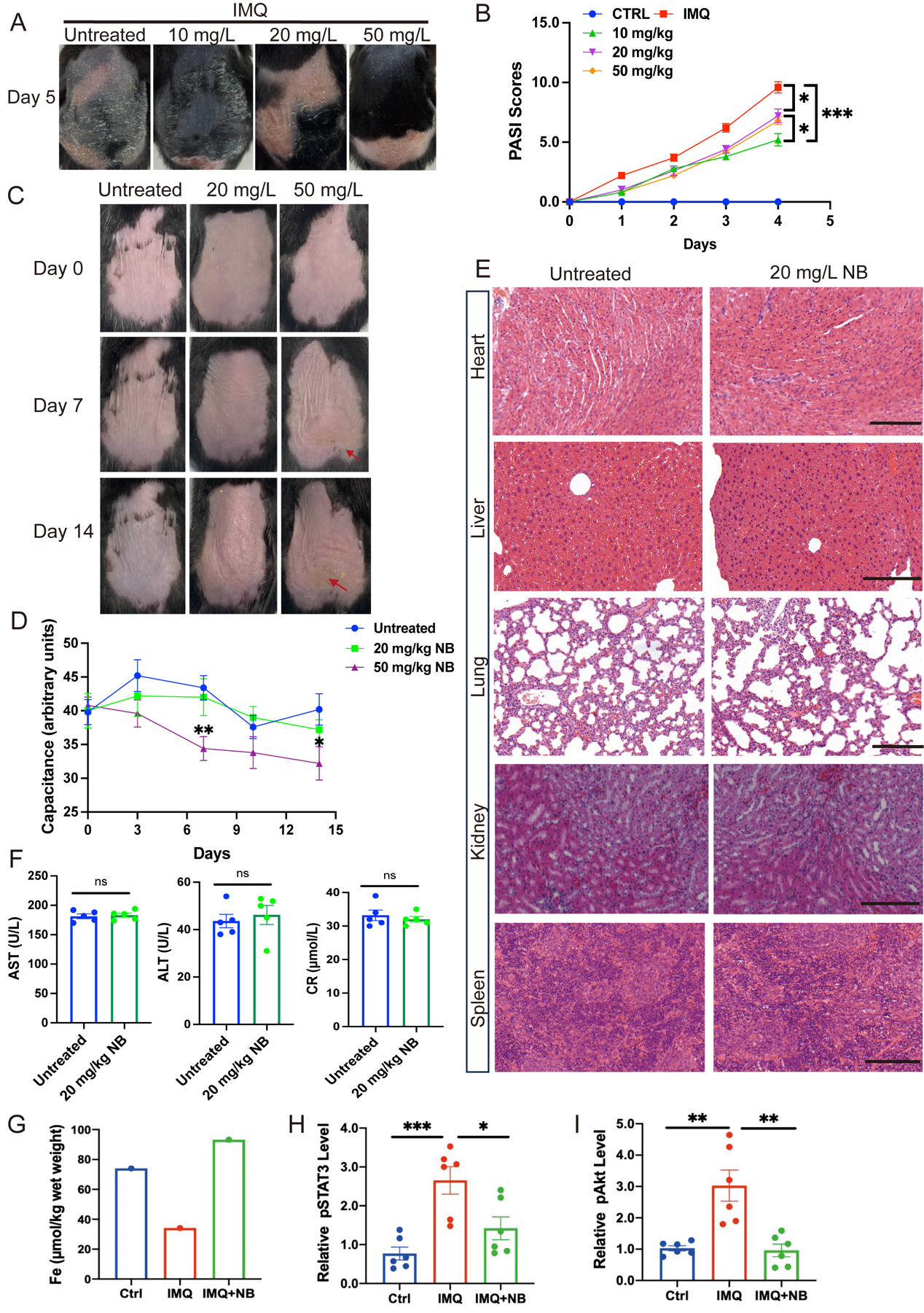
***

**Figure S9. A)** Representative images of 10 mg L^-1^, 20 mg L^-1^ and 50 mg L^-1^ NB on day 5 under IMQ stimulation. **B)** PASI of four intervention groups *vs.* CTRL. **C)** Representative images of continuous injection of 20 mg L^-1^ and 50 mg L^-1^ NB on day 0, day 7 and day 14. **D)** Moisture content of stratum corneum. **E****)** H & E staining of heart, liver, lung, kidney and spleen. Scale bar = 200 μm. **F)** CR, AST and ALT test. *n* = 5 mice per group. **G)** Calculated Fe (μmol kg^-1^ wet weight) through OD 593 nm. **H-I)** Quantification results of the immunoblotting. (Two-tailed Student’ s t test was used for the statistical analysis. Data are presented as means ± SEM (*n* = 5 mice per group). **p* < 0.05, ***p* < 0.01, ****p* < 0.001, ns, no significance)

***Supplementary Figure S10***


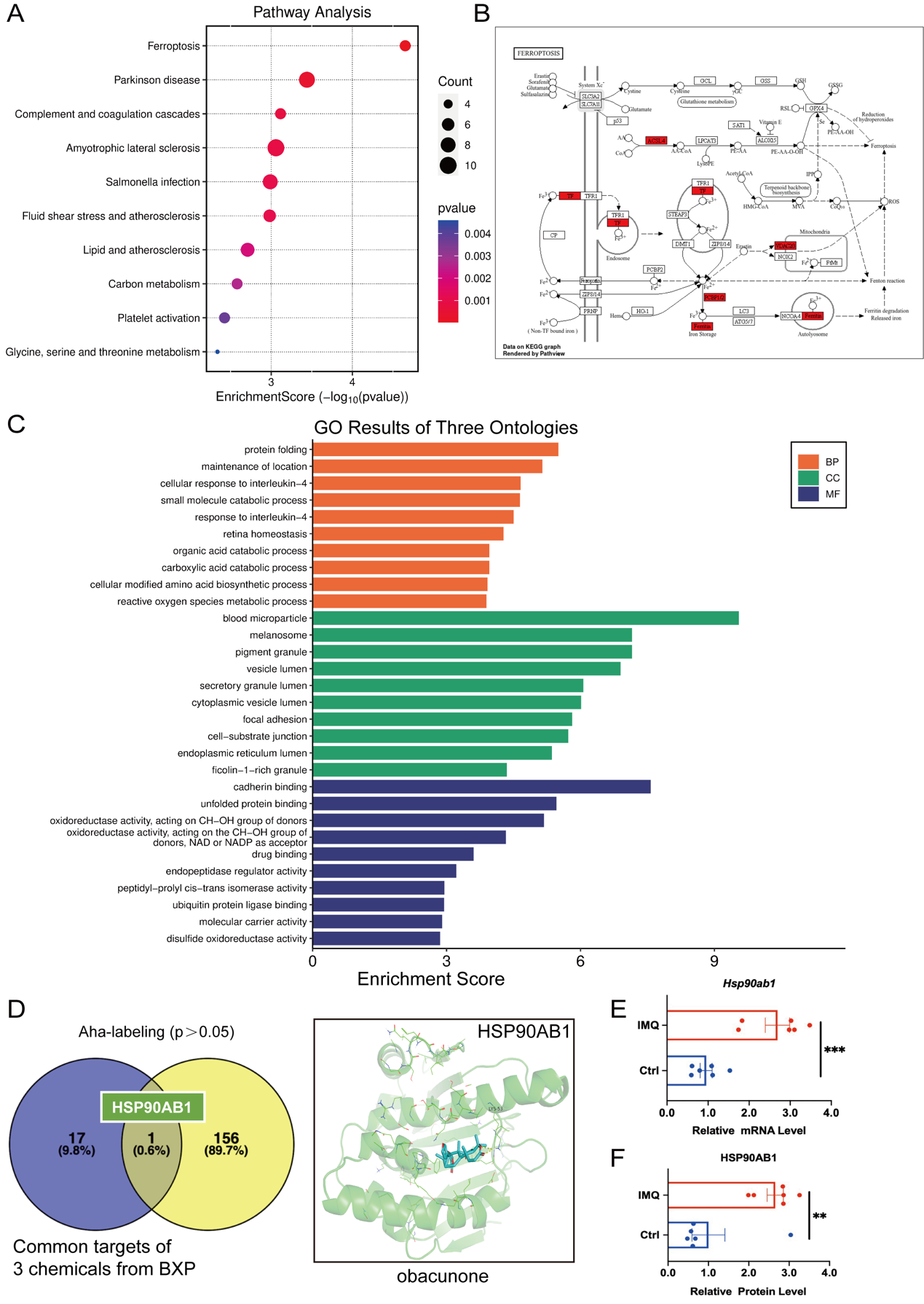


**Figure S10. A)** Enrichment of the pathway from NB *vs.* Ctrl. **B)** Hsa04216 Ferroptosis pathway. **C)** GO results of three ontologies. **D)** Venn diagram of 156 significant changed proteins and common target lists of three chemicals from BXP，and molecular docking of obacunone with HSP90AB1. **E-F)** Quantification results of (**E**) q-PCR and (**F**) the immunoblotting of HSP90AB1. (Two-tailed Student’ s t test was used for the statistical analysis. Data are presented as means ± SEM. ***p* < 0.01, ****p* < 0.001)

***Supplementary Figure S11 Quantification Results***


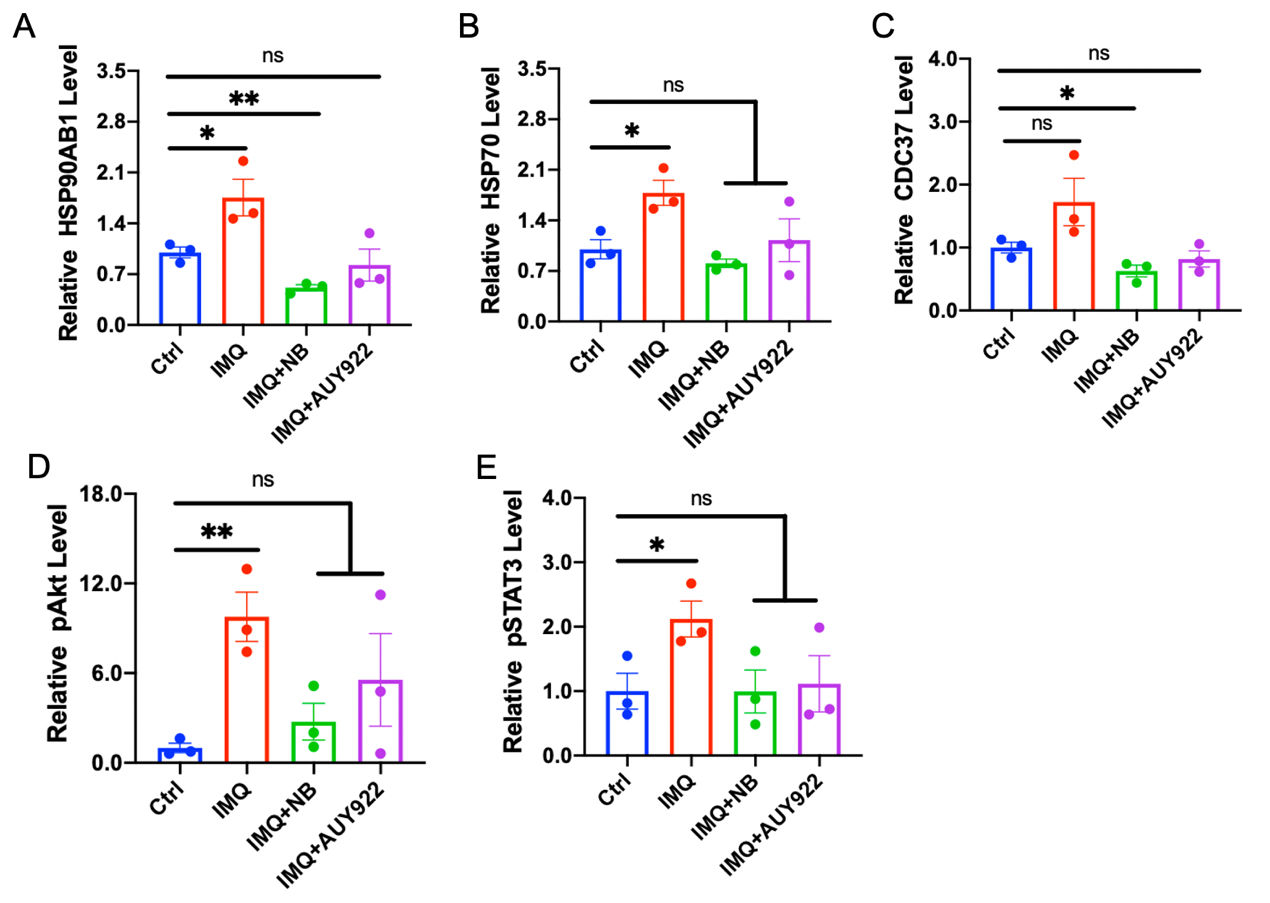


**Figure S11.** **Quantification results.** Quantification results of the immunoblotting of HSP90AB1 (**A**), HSP70 (**B**), CDC37 (**C**), p-Akt (**D**) and p-STAT3 (**E**). (Two-tailed Student’ s t test was used for the statistical analysis. Data are presented as means ± SEM (*n* = 3 mice per group). **p* < 0.05, ***p* < 0.01, ns, no significance)

***Supplementary Figure S12*** ***Predicted Transcription Factors of HSP90AB1 and The Correlation Analysis***


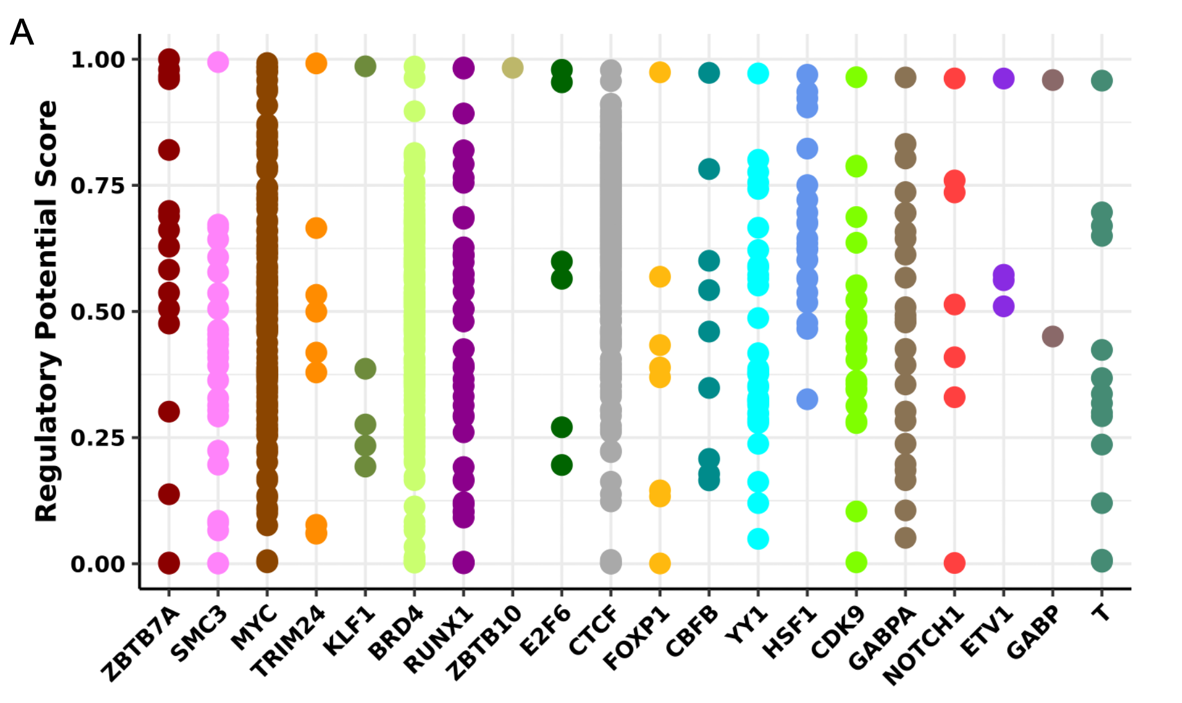

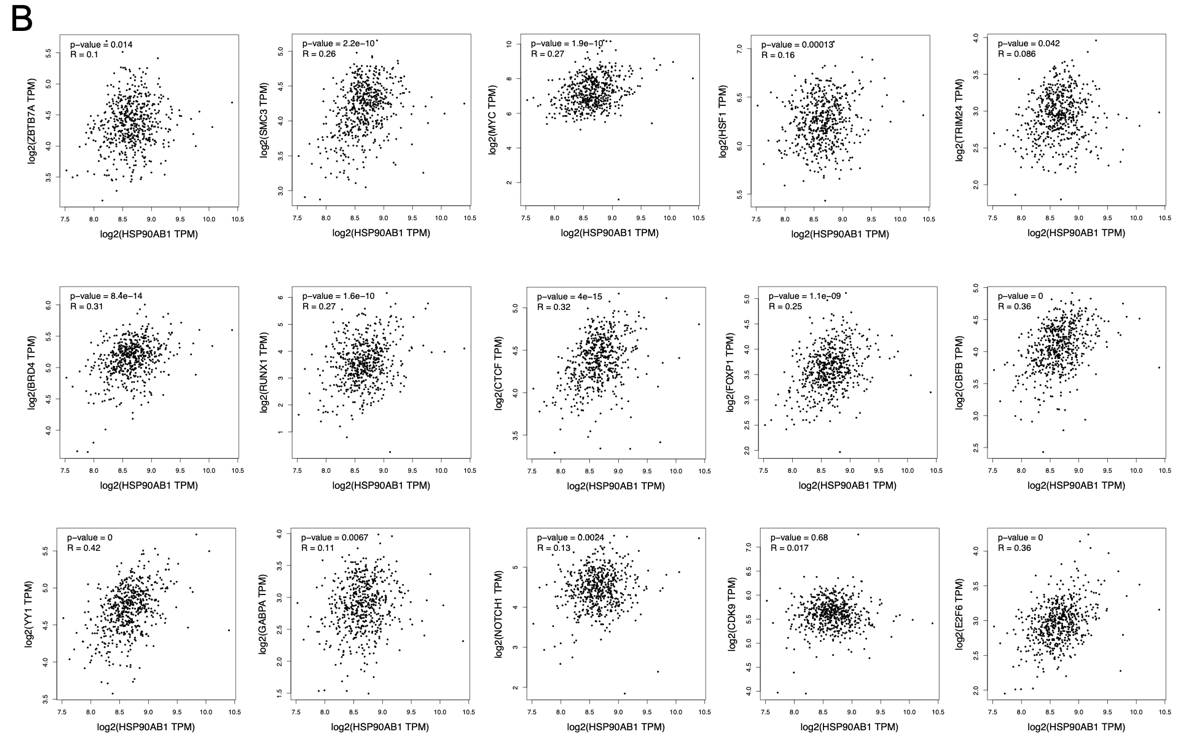


**Figure S12. Predicted transcription factors of HSP90AB1 and the correlation analysis. A)** Top 20 factors are showed in the plot. Y axis represents the RP score. X axis represents different factors. Dots in a x axis line means same factor. **B)** Correlation analysis (Pearson) of HSP90AB1 and factors basing on GEPIA-GTEx (Skin-Not Sun Exposed and Skin-Sun Exposed).

***Supplementary Figure S13 Enriched Peak Analysis of Control and NB***


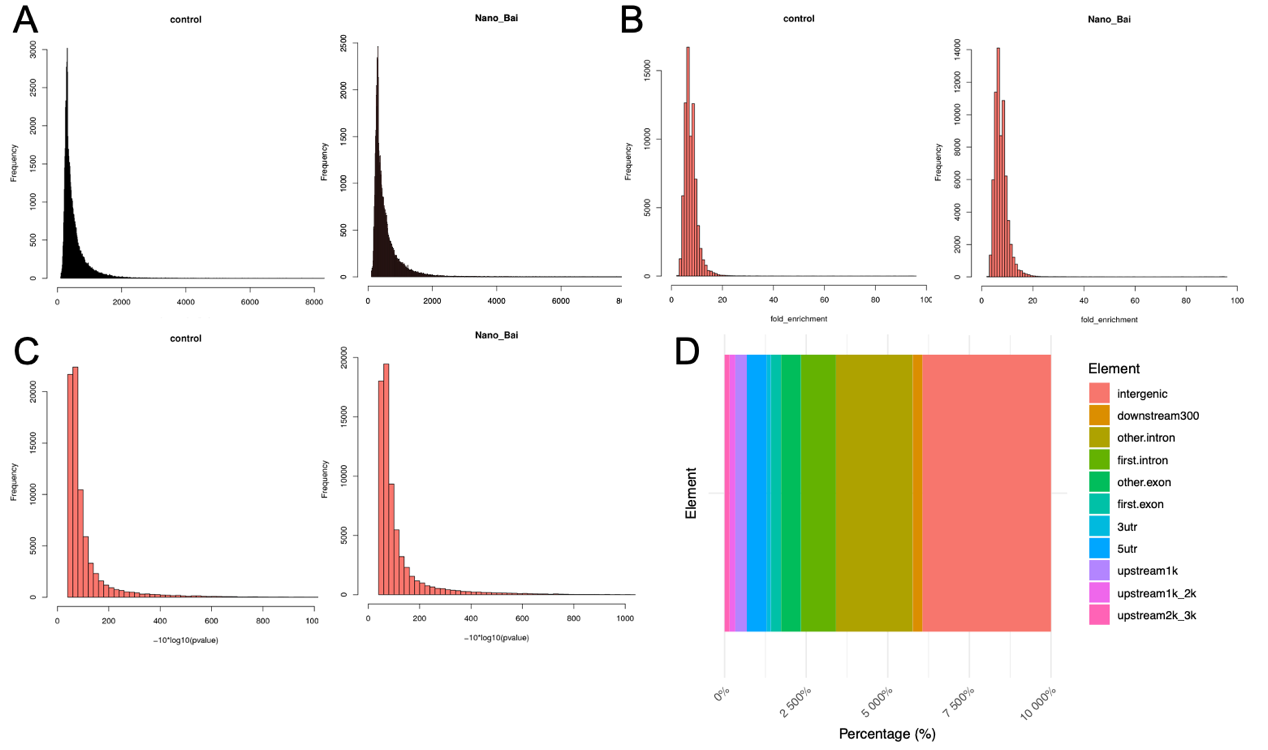


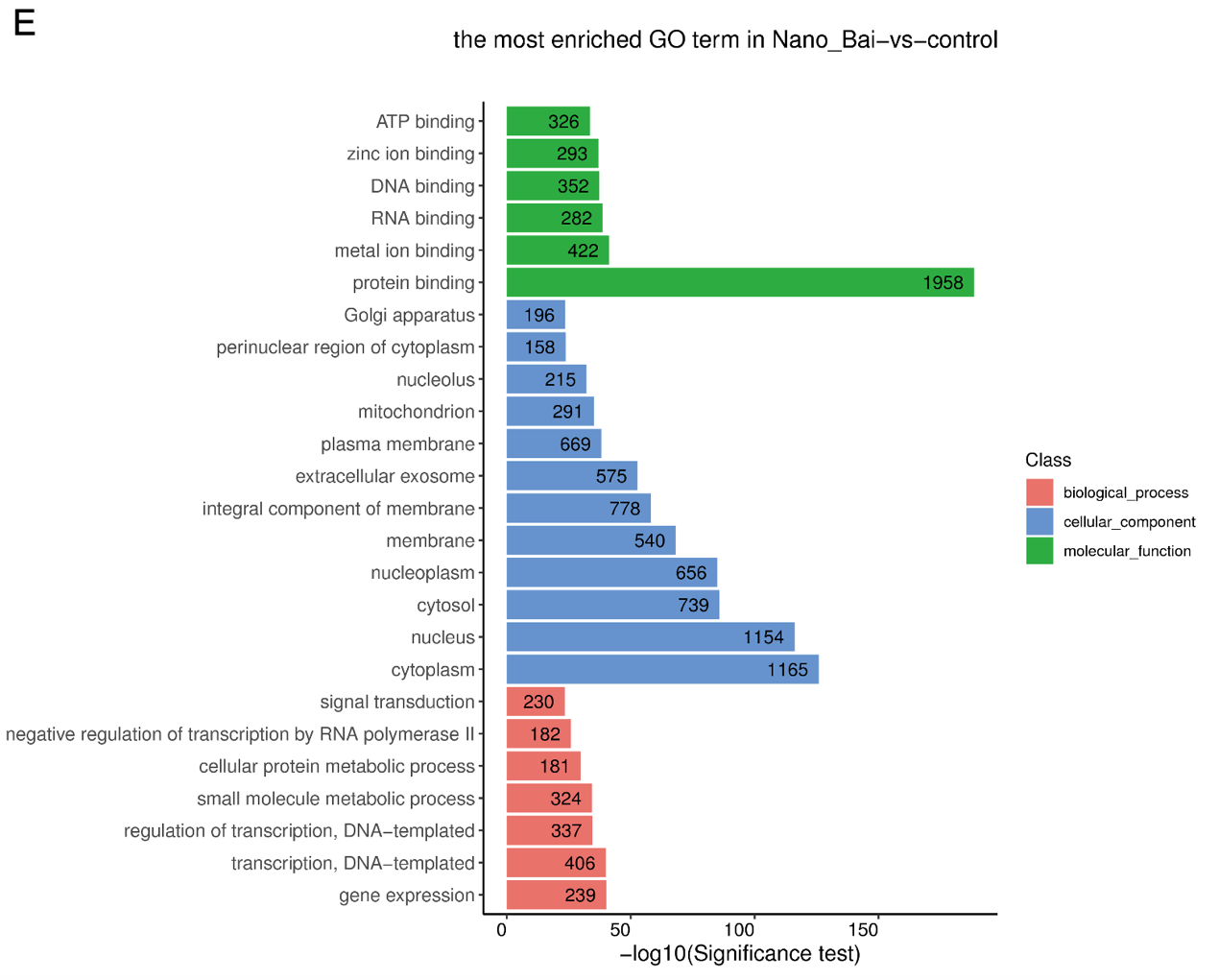


**Figure S13. Enriched peak analysis of Control and NB. A)** The length of the peak. **B)** Signal Value. **C)** q value of each peak. **D)** The element distribution. **E)** The most enriched GO term in NB *vs.* Ctrl.

***Supplementary Figure S14 IGV Visual Analysis of Other Transpiration Factors in NB and Control Group***

***
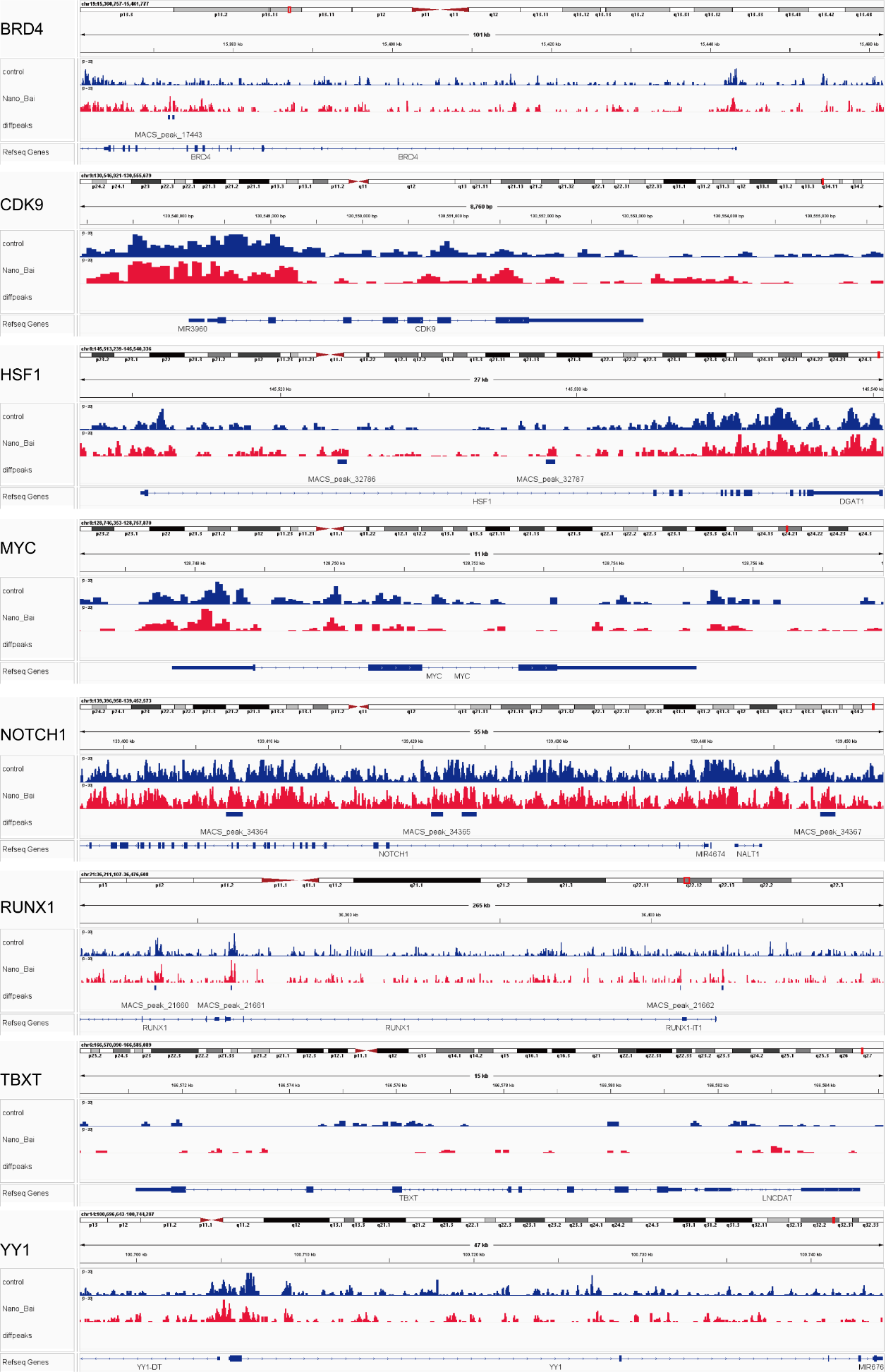
***

**Figure S14. IGV visual analysis of other transpiration factors** **in NB and Control group.**

***Supplementary Figure S15 Quantification Results and Immunofluorescence of AUY922 Group***

***
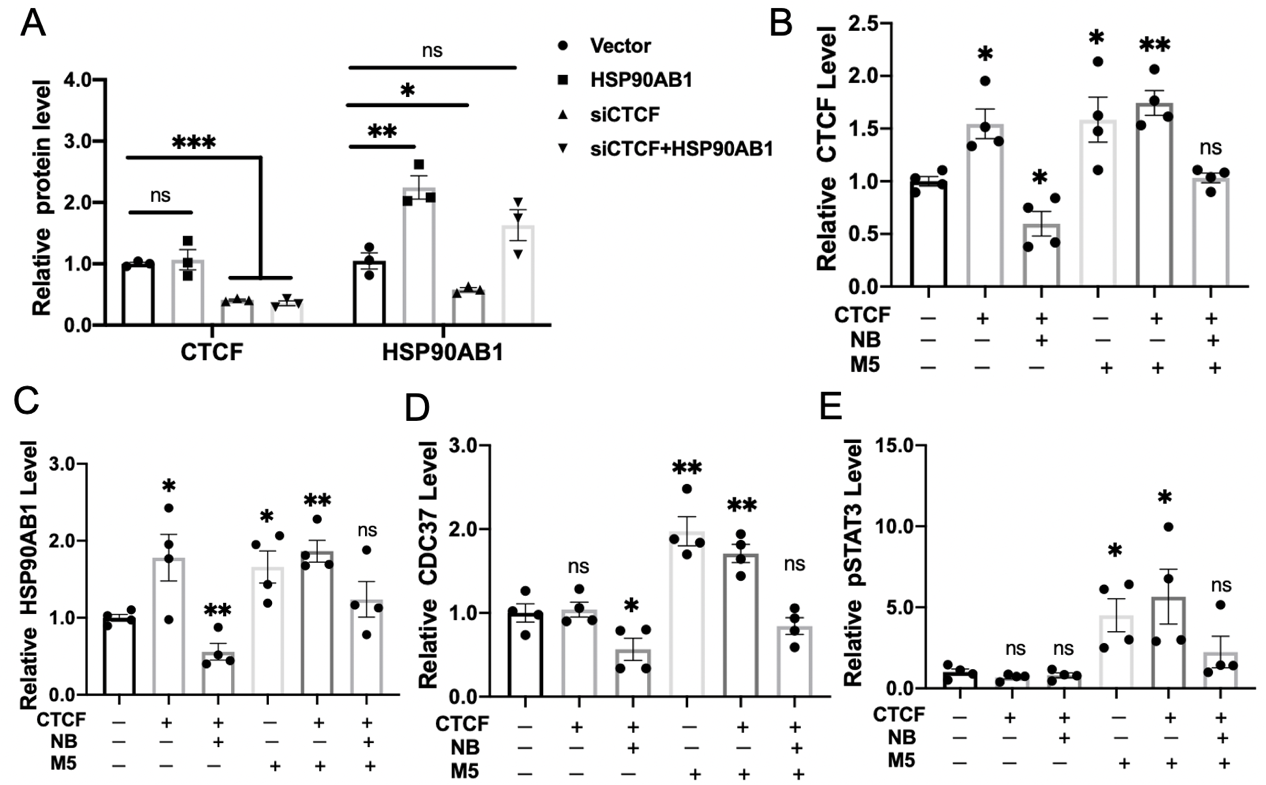

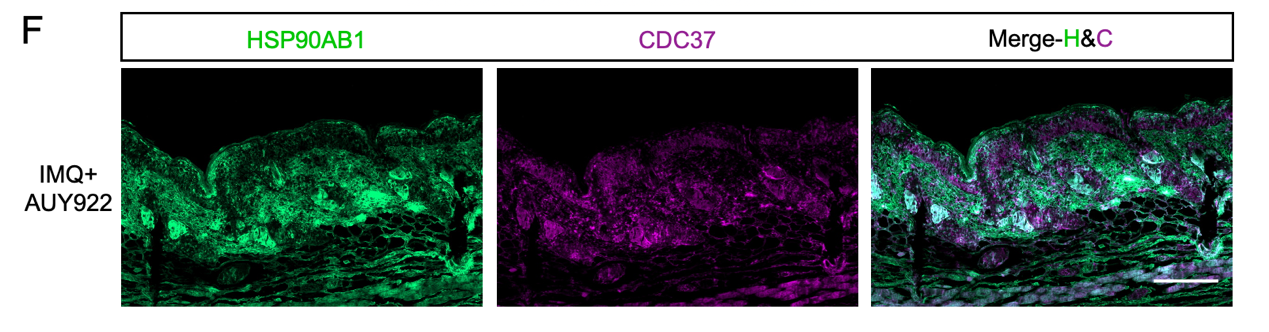
***

**Figure S15. Quantification results and immunofluorescence of AUY922 group.** Quantification results of the immunoblotting of CTCF and HSP90AB1 (**A**) with transfection of siCTCF and *HSP90AB1* overexpression plasmids. Quantification results of the immunoblotting of CTCF (**B**), HSP90AB1 (**C**), CDC37 (**D**), and p-STAT3 (**E**) with transfection of *CTCF* overexpression plasmids and with/without M5/NB treatment. **F)** Immunofluorescence of HSP90AB1-CDC37 from sections of IMQ mice with AUY922 treatment (*n* = 3 mice per group). (Two-tailed Student’ s t test was used for the statistical analysis. Data are presented as means ± SEM (*n* = 3 or 4 biologically independent cell samples). **p* < 0.05, ***p* < 0.01, ****p* < 0.001, ns, no significance)

**Reference**

[1] Y. Huang, C. Zhao, G. Zheng, Y. Yuan, L. Gong, R. Liu, J. An, Dictamnine Ameliorates DNFB-Induced Atopic Dermatitis Like Skin Lesions in Mice by Inhibiting M1 Macrophage Polarization and Promoting Autophagy, *Biol Pharm Bull* **2024**, 47, 175.

[2] O. G. Best, S. P. Mulligan, Heat shock protein-90 inhibitor, NVP-AUY922, is effective in combination with fludarabine against chronic lymphocytic leukemia cells cultured on CD40L-stromal layer and inhibits their activated/proliferative phenotype, *Leuk. Lymphoma* **2012**, 53, 2314.

[3] L. van der Fits, S. Mourits, J. S. A. Voerman, M. Kant, L. Boon, J. D. Laman, F. Cornelissen, A. M. Mus, E. Florencia, E. P. Prens, E. Lubberts, Imiquimod-Induced Psoriasis-Like Skin Inflammation in Mice Is Mediated via the IL-23/IL-17 Axis, *J. Immunol.* **2009**, 182, 5836.

[4] a) J. C. Farfán-Esquivel, M. V. Gutiérrez, A. Ondo-Méndez, J. M. González, M. J. Vives-Flórez, Antibacterial activity and impact on keratinocyte cell growth of Cutibacterium acnes bacteriophages in a Cutibacterium acnes IA(1)- colonized keratinocyte model, *Curr Res Microb Sci* **2025**, 8, 100356; b) C. Li, L. Xiao, J. Jia, F. Li, X. Wang, Q. Duan, H. Jing, P. Yang, C. Chen, Q. Wang, J. Liu, Y. Shao, N. Wang, Y. Zheng, Cornulin Is Induced in Psoriasis Lesions and Promotes Keratinocyte Proliferation via Phosphoinositide 3-Kinase/Akt Pathways, *J. Invest. Dermatol.* **2019**, 139, 71.

[5] Z. Zhang, B. Cheng, W. Du, M. Zeng, K. He, T. Yin, S. Shang, T. Su, D. Han, X. Gan, Z. Wang, M. Liu, M. Wang, J. Liu, Y. Zheng, The Role of Nicotinamide Mononucleotide Supplementation in Psoriasis Treatment, *Antioxidants* **2024**, 13, 186.

[6] Y. Hao, T. Stuart, M. H. Kowalski, S. Choudhary, P. Hoffman, A. Hartman, A. Srivastava, G. Molla, S. Madad, C. Fernandez-Granda, R. Satija, Dictionary learning for integrative, multimodal and scalable single-cell analysis, *Nat. Biotechnol.* **2024**, 42, 293.

[7] K. Street, D. Risso, R. B. Fletcher, D. Das, J. Ngai, N. Yosef, E. Purdom, S. Dudoit, Slingshot: cell lineage and pseudotime inference for single-cell transcriptomics, *BMC Genomics* **2018**, 19, 477.

[8] A. T. Feldman, D. Wolfe, Tissue processing and hematoxylin and eosin staining, *Methods Mol. Biol.* **2014**, 1180, 31.
